# BMS1 Is Mutated in Aplasia Cutis Congenita

**DOI:** 10.1371/journal.pgen.1003573

**Published:** 2013-06-13

**Authors:** Alexander G. Marneros

**Affiliations:** Cutaneous Biology Research Center, Massachusetts General Hospital, Department of Dermatology, Harvard Medical School, Charlestown, Massachusetts, United States of America; Stowers Institute for Medical Research, United States of America

## Abstract

Aplasia cutis congenita (ACC) manifests with localized skin defects at birth of unknown cause, mostly affecting the scalp vertex. Here, genome-wide linkage analysis and exome sequencing was used to identify the causative mutation in autosomal dominant ACC. A heterozygous Arg-to-His missense mutation (p.R930H) in the ribosomal GTPase BMS1 is identified in ACC that is associated with a delay in 18S rRNA maturation, consistent with a role of BMS1 in processing of pre-rRNAs of the small ribosomal subunit. Mutations that affect ribosomal function can result in a cell cycle defect and ACC skin fibroblasts with the BMS1 p.R930H mutation show a reduced cell proliferation rate due to a p21-mediated G1/S phase transition delay. Unbiased comparative global transcript and proteomic analyses of ACC fibroblasts with this mutation confirm a central role of increased p21 levels for the ACC phenotype, which are associated with downregulation of heterogenous nuclear ribonucleoproteins (hnRNPs) and serine/arginine-rich splicing factors (SRSFs). Functional enrichment analysis of the proteomic data confirmed a defect in RNA post-transcriptional modification as the top-ranked cellular process altered in ACC fibroblasts. The data provide a novel link between BMS1, the cell cycle, and skin morphogenesis.

## Author Summary

Elucidating the pathomechanisms in congenital diseases of the skin provides the opportunity to learn what cellular processes are important during embryonic development of the skin structures. Aplasia cutis congenita (ACC) manifests with localized skin defects, most commonly affecting the scalp skin. Here, global proteomic and transcriptional analyses are combined with genome-wide linkage and exome sequencing approaches to identify the molecular mechanisms involved in ACC. A mutation in the ribosomal GTPase BMS1 is identified in ACC that affects 18S rRNA maturation. This mutation is associated with a p21-mediated G1/S phase transition delay during the cell cycle that inhibits cell proliferation. The findings are consistent with mutations in ribosomal disorders that result in nucleolar stress and a G1/S phase transition delay. Thus, mutations in BMS1 can affect the formation of a highly proliferative tissue during development, such as the rapidly expanding scalp epidermis.

## Introduction

Aplasia cutis congenita (ACC [MIM 107600]) manifests at birth as a localized skin defect that usually heals with a hypertrophic scar. Most commonly the scalp skin is affected and results in localized alopecia at the site of the defect, but sometimes the defect can extend into deeper structures involving the dura mater or osseous structures. ACC has to be distinguished clinically from birth trauma or intrauterine herpetic infections. Most reported cases are sporadic, but autosomal dominant inheritance has been reported as well [1]. A causative gene mutation for non-syndromic ACC has not been reported so far. While the majority of patients with aplasia cutis have no other congenital abnormalities, aplasia cutis can occur as part of rare syndromes with a wide spectrum of anomalies. For example, some patients with Johanson-Blizzard syndrome [MIM 243800] have been reported to have aplasia cutis of the scalp skin. This syndrome is characterized by nasal alar hypoplasia, hypothyroidism, pancreatic achylia and congenital deafness and is caused by a mutation in the *UBR1* gene [2]. Adams-Oliver syndrome (AOS [MIM 100300]) is characterized by ACC and transverse limb defects, but a wide range of additional congenital anomalies have been reported in patients with AOS, including congenital heart defects. Recently gain-of-function mutations in the *ARHGAP31* gene encoding a Cdc42/Rac1 regulatory protein have been reported in AOS [3], and two more cases of AOS were reported to harbor homozygous mutations in the *DOCK6* gene, encoding a guanidine nucleotide exchange factor that activates Cdc42 and rac1 [4]. A recent report showed mutations in the transcriptional regulator of the Notch pathway, *RBPJ*, in two small families with AOS [5]. Furthermore, mutations in the *KCTD1* gene were recently reported in patients with Scalp-Ear-Nipple syndrome, which manifests with scalp ACC lesions as well [6]. However, the majority of individuals with aplasia cutis have no other congenital anomalies, suggesting a distinct pathomechanism between non-syndromic ACC and syndromic cases as in AOS or other syndromes in which aplasia cutis has been described.

Histologically, ACC shows in most cases a complete absence of epidermis at birth suggesting a skin morphogenesis defect. It has been speculated that a defect in cell proliferation may be underlying ACC that may lead to a delay in skin closure ability late during development at an anatomic site where a steady expansion of the brain structures may require a rapid proliferation of the overlying skin. However, neither the genetic basis for ACC nor the pathomechanisms that result in the skin formation defects are known. Identifying the genetic causes for ACC and determining their molecular consequences promises to reveal new mechanisms that govern skin morphogenesis during development. Here, by combining genome-wide linkage analysis with exome sequencing approaches, a mutation in the ribosomal GTPase BMS1 is identified in autosomal dominant ACC that is associated with a p21-mediated G1/S phase cell cycle transition delay and results in a reduced cell proliferation rate. In addition, unbiased expression profiling and proteomic analyses in fibroblasts carrying this mutation independently confirm a central role of a p21-mediated G1/S phase cell cycle delay for the ACC phenotype.

## Results

### A mutation in the ribosomal GTPase BMS1 causes autosomal-dominant ACC

**Figure 1:**
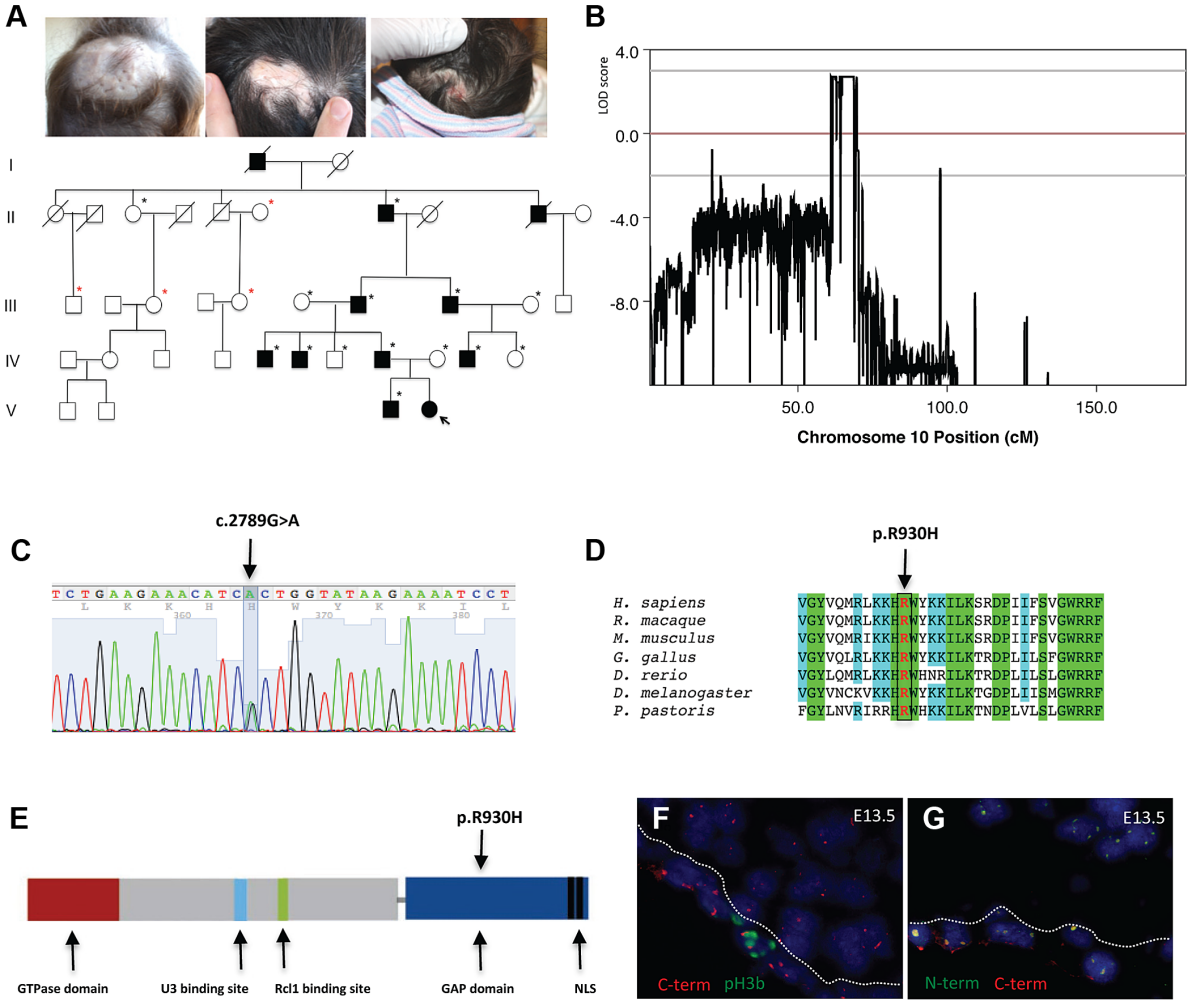
A mutation in the ribosome biogenesis GTPase BMS1 causes autosomal-dominant ACC. a. Representative images of individuals with ACC in a five-generation pedigree with autosomal dominant inheritance pattern and full penetrance. (Left image: 1 year old with hypertrophic scar at site of congenital ACC of the scalp; Middle image: father of 1 year old of left image with scar at site of congenital ACC; Right image: daughter of this father on day 1 after birth with localized erosion on vertex of scalp (indicated with arrow in pedigree; this member was born after the linkage study was completed and carried the disease allele as well)). Family members' DNA used for genome-wide SNP genotyping indicted with black stars; additional family members' DNA included for confirmation of linkage with microsatellite markers indicated in red stars. b. Histogram of multi-point LOD scores along chromosome 10 shows a single chromosomal region with LOD scores >2 on chromosome 10q11. c. A heterozygous missense mutation resulting in a G>A nucleotide change in the BMS1 gene was identified in all affected members with ACC in this family (c.2789G>A). d. The c.2789G>A mutation results in a Arg-to-His amino acid change (p.R930H) of a conserved Arg residue in BMS1. e. The p.R930H amino acid change affects an Arg residue within the putative C-terminal GAP domain of BMS1. Representative image of the domains of BMS1 with its GTPase domain at the N-terminus (adapted from [9]). f. Murine embryonic sections showing the site of skin formation at the vertex area of the scalp at E13.5 (f. and g.). BMS1 expression is seen in all cells, including the proliferative epidermis (C-terminus of BMS1 in red, pH3b green). C-term indicates labeling with the monoclonal anti-BMS1 antibody that recognizes the C-terminus of BMS1. pH3b indicates staining for the proliferation marker phospho-Histone 3B (Ser10) (pH3b, green). g. Co-localization of BMS1 C-terminal (C-term red) and N-terminal domains (N-term green) at E13.5 of embryonic murine scalp at E13.5. Nuclei are labeled with DAPI. All images are acquired with a 20× objective.

In this study, a five-generation family with autosomal dominant inheritance of ACC was identified (Fig. 1a). In this family the exclusive congenital anomaly is a localized absence of skin at the vertex and occipital area of the scalp that most often healed with a hypertrophic scar (Fig. 1a). No other congenital anomalies were identified, excluding rare syndromes that can manifest with ACC [2], [3], [4].

Genome-wide linkage analysis revealed a single genomic region with a LOD score>2 on chromosome 10q11. The maximal LOD score of 2.709 was reached with a sharp peak between marker rs1359280 located at 34,877,513 bp and marker rs7071514 located at 49,518,113 bp (Fig. 1b) (an additional affected family member who carried the disease allele was born after the linkage study was completed, which further increased the LOD score). Haplotypes and recombination sites were independently confirmed using microsatellite markers. Whole-exome sequencing was performed in one affected individual of this family. Sequencing of coding regions was performed to a mean coverage of 151× to generate 17.3 Gb of sequence. Variants were filtered to exclude homozygous base pair changes, non-coding variants, synonymous variants and all non-synonymous changes that are present in dbSNP129, the 1000genomes database and the NHLBI Exome Sequencing Project (ESP) database. This genome-wide sequencing approach resulted in the identification of a single non-synonymous heterozygous G>A base change within the linked genomic region. This sequence change was not identified in 5,351 unrelated individuals for which high-quality sequence calls were made at this position in the ESP sequence data, suggesting that the G>A sequence change is not a rare variant but pathogenic in this family with ACC. Bidirectional Sanger sequencing was performed in all members of this family, showing that none of the unaffected family members had the G>A sequence change, but all affected family members had this sequence change, thus co-segregating with the disease allele. Finally, sequencing of DNA from 100 geographically and ethnically matched unaffected control individuals did not reveal this mutation as well.

This heterozygous G>A mutation results in an Arg-to-His amino acid change at position 930 (p.R930H) of a conserved Arginine within the C-terminal domain of the ribosome assembly GTPase BMS1, which has previously not been implicated in skin morphogenesis (Figure 1c–1e). BMS1 is a component of the U3 snoRNA-containing complex that has been shown to be essential in yeast and to be conserved in eukaryotes. Depletion of BMS1 interferes with pre-ribosomal RNA (rRNA) processing at sites A_0_, A_1_ and A_2_ and the formation of the 40S ribosomal subunit [7], [8]. Biochemical analyses demonstrated that BMS1 may function as a GTPase, with its GTPase activity located at the N-terminus of the protein. In addition to binding to U3 snoRNA, BMS1 binds the endonuclease Rcl1 in a GTP-dependent manner and BMS1 binding is required to recruit Rcl1 to preribosomes. Thus, it has been suggested that BMS1 functions as a GTP-regulated switch to deliver Rcl1 to preribosomes and has an essential role in the formation of the small ribosomal subunit [9], [10]. The C-terminal domain of BMS1 has been proposed to act as a putative intramolecular GTPase-activating protein (GAP) domain and is separated from the rest of the protein by a flexible linker region [9]. However, the in vivo consequences of an impairment of this putative GAP domain for cellular functions have not been defined, and the BMS1 p.R930H mutation in ACC provides the first indication for a role of this domain for overall BMS1 function in vivo.

### ACC skin fibroblasts with the BMS1 p.R930H mutation show a delay in pre-rRNA processing

**Figure 2:**
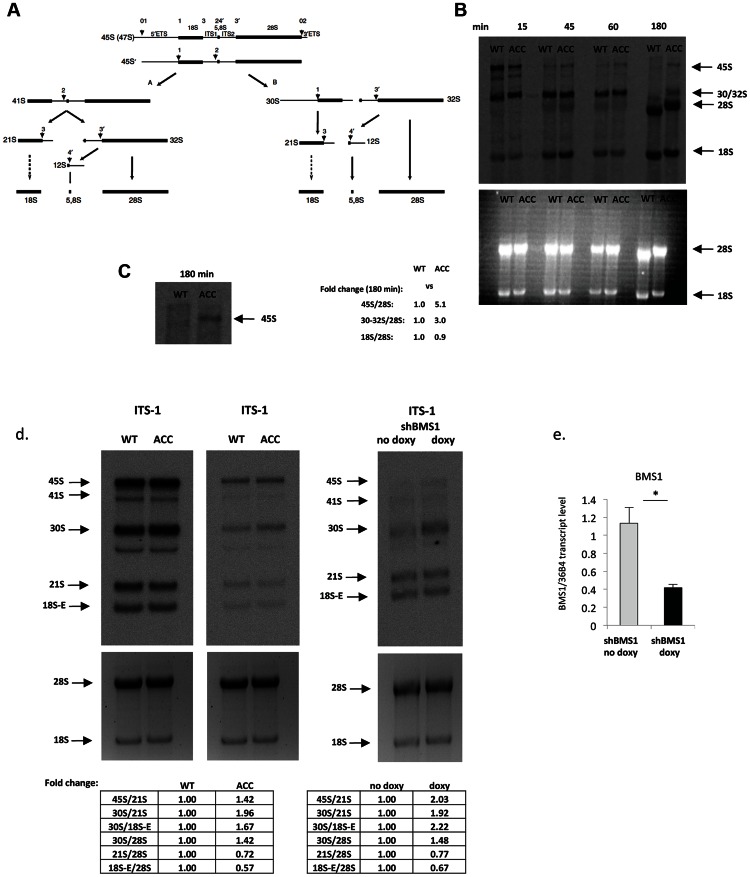
ACC fibroblasts with mutated BMS1 show a delay in 18S pre-rRNA processing. a. Pre-rRNA processing pathways in human cells (adapted from [33]). Two pathways coexist depending on the order of cleavage in the 5′-ETS (sites A_0_ and A_1_) and ITS1 (A_2_). Nomenclature of the cleavage sites follows Hadjiolova et al. (1993) and Rouquette et al. (2005) [33], [34]. b. Pulse-chase labeling of pre-rRNAs with L-[*methyl*-^3^H]methionine shows that ACC^BMS1(p.R930H)^ fibroblasts (ACC) form all pre-rRNA species compared to control fibroblasts (WT), but retain increased 45S and 30S pre-rRNAs (seen at 180 minutes after pulse-labeling). Relative values for bands normalized to 28S rRNA band are shown (fold-change compared to WT). c. Magnification of 45S pre-rRNAs 180 minutes after pulse labeling shows retained 45S pre-rRNA in ACC^BMS1(p.R930H)^ fibroblasts compared to control fibroblasts (WT). d. Northern blot analysis of pre-rRNA processing using a radioactive ITS-1 probe. RNA from ACC fibroblasts was used carrying the BMS1 p.R930H mutation, as well as from control fibroblasts (WT). The Northern blot shown on the left side for the WT and ACC cells was exposed overnight, while the image on the right side shows the same blot after a 4 hour exposure. In addition, RNA from fibroblasts stably transfected with an inducible BMS1 shRNA vector was used, after shRNA-mediated knockdown of BMS1 transcripts was induced by doxycycline (doxy) treatment. Ethidiumbromide stained gels prior to blotting (bottom) confirm equal loading of RNA. Quantitation of band intensity ratios expressed as relative values (fold-change compared to WT or untreated cells). e. Doxycyline-inducible knockdown of BMS1 by shRNA lentivirus (shBMS1). Doxycycline treatment for 48 hours results in ∼60% reduction of BMS1 transcript levels. * P-value<0.05.

BMS1 is ubiquitously expressed, including the proliferative developing skin of the scalp that is affected in ACC (Fig. 1f, 1g). The BMS1 p.R930H mutation within the putative GAP domain does not affect BMS1 expression or its nucleolar localization (Fig. S1). Due to the heterozygous nature of the mutation within a regulatory domain of BMS1 with a remaining wild-type allele and the only localized congenital defect in ACC, it would be expected that the p.R930H mutation in BMS1 leads to rather modest abnormalities in pre-rRNA processing and therefore results in a restricted phenotype. Indeed, pulse-chase labeling experiments with ACC^BMS1(p.R930H)^ fibroblasts (isolated from an affected member of this family) and with matched unrelated wild-type fibroblasts, in which labeling of pre-rRNAs was performed with L-[*methyl*-^3^H]methionine, showed formation of all pre-rRNAs but with higher levels of persistent 45S pre-rRNA and the pre-rRNA band likely corresponding to the 30S pre-rRNA in ACC^BMS1(p.R930H)^ fibroblasts (Fig. 2a–2c). Northern blotting experiments using RNA from ACC^BMS1(p.R930H)^ fibroblasts and control fibroblasts with probes for ITS-1, ETS-1 and ITS-2 (transcribed spacers) showed an increase of 45S and 30S pre-rRNAs in mutant cells and a relative decrease of 21S and 18S-E pre-rRNAs (precursors for 18S rRNA of the small ribosomal subunit), while 32S and 12S pre-rRNAs (precursors for 5.8S and 28S rRNAs of the large ribosomal subunit) were not affected (Fig. 2d, Fig. S2). The findings of the pulse-chase labeling and Northern blotting experiments in ACC^BMS1(p.R930H)^ fibroblasts are consistent with a delay in pre-rRNA processing affecting the small ribosomal subunit, as would be predicted based on the reported role of BMS1 for the small ribosomal subunit processome in yeast [7], [8].

Similarly, inducible shRNA-mediated stable knockdown of BMS1 to achieve ∼40–50% of BMS1 transcript levels (to correspond to heterozygous BMS1 cells) resulted also in a relative increase of 45S and 30S pre-rRNAs, and a relative decrease in 21S and 18S-E pre-rRNAs, while 32S pre-rRNAs were reduced less when compared to the 30S pre-rRNAs (Fig. 2d and Fig. S2). These findings suggest that the BMS1 p.R930H mutation results in a reduced activity of BMS1 during the processing of 18S pre-rRNAs.

### A p21-mediated G1/S phase transition defect in ACC skin fibroblasts with the BMS1 p.R930H mutation

Mutations in several genes that affect ribosomal function have been described to result in a G1/S phase transition delay that may involve p53-dependent and p53-independent pathways, presumably due to “nucleolar stress” resulting from increased free ribosomal proteins as a consequence of ribosomal processing or assembly abnormalities [11]. To determine whether ACC fibroblasts that carry the BMS1 p.R930H mutation show a similar cellular response as in some ribosomopathies, these fibroblasts were examined for their proliferation rate and whether a G1/S phase transition delay can be observed.

**Figure 3:**
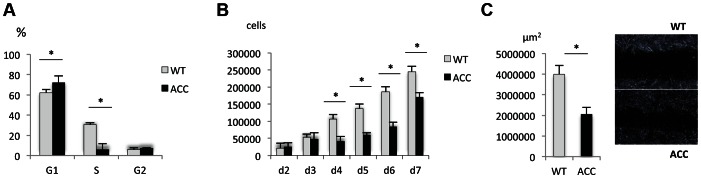
ACC fibroblasts with mutated BMS1 show a G1/S phase cell cycle transition delay and reduced cell proliferation rate. a. Primary ACC fibroblasts show a significantly reduced proportion of cells in S-phase compared to control fibroblasts (WT), consistent with a delay in G1/S phase transition in mutant cells. PI staining and FACS-based analysis. * P-value<0.05 for cells in G1 and S phase. b. Cell proliferation rate is reduced in ACC fibroblasts. * P-value<0.05 for values between d4 to d7. c. In vitro scratch assay of fibroblasts on fibronectin-coated dishes show increased cell migration rate in ACC fibroblasts (assayed at 14 hours after scratch). Images taken with a 2.5× objective. * P-value<0.05. Graph shows area not repopulated with fibroblasts.

Skin fibroblasts were isolated from an affected family member through a skin biopsy and expanded in vitro. Control fibroblasts were obtained from an unrelated unaffected individual, matched for ethnicity, age and anatomic location. Subconfluent early-passage ACC fibroblasts and control fibroblasts were analyzed for their cell cycle status, cell proliferation rate and cell migration ability. FACS-based cell cycle analysis showed a G1/S phase transition defect in ACC fibroblasts (Fig. 3a), with a significantly reduced cell proliferation rate (Fig. 3b). Furthermore, cell migration rate was increased in ACC fibroblasts in an in vitro scratch assay, which is consistent with the observation that cell migration is favored in G1 phase (Fig. 3c) [12].

Thus, similarly as has been observed in some ribosomopathies, ACC fibroblasts with the BMS1 p.R930H mutation show a link between a mutation in a gene involved in ribosomal function and a G1/S phase transition delay that results in a reduced cell proliferation rate.

To determine the molecular changes that are associated with a G1/S phase transition defect and a reduced cell proliferation rate in ACC, unbiased analyses of primary ACC^BMS1(p.R930H)^ fibroblasts compared to control fibroblasts were performed using global gene expression profiling and quantitative comparative proteomic experiments.

### Global transcript and proteomic analyses in ACC^BMS1(p.R930H)^ skin fibroblasts

**Figure 4:**
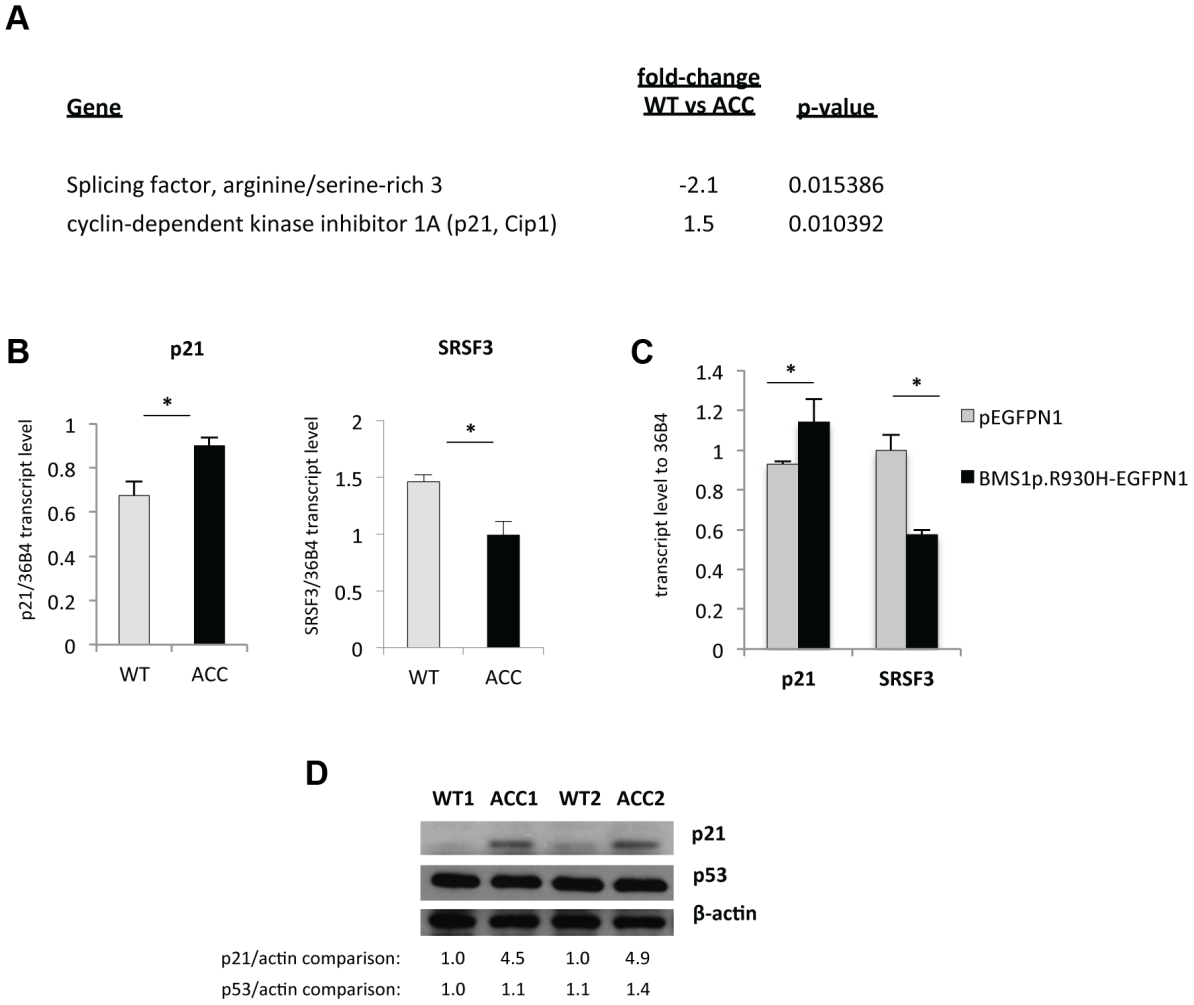
An increase in p21 levels in ACC skin fibroblasts. a. Microarray gene expression profiling experiments (experimental triplicates for ACC vs control fibroblasts) show an upregulation of p21 mRNA levels and a downregulation of SRSF3 mRNA levels in ACC fibroblasts. b. These findings were confirmed by semiquantitative RT-PCR. *P-values<0.05. c. Overexpression of mutant BMS1p.R930H in wild-type fibroblasts phenocopies ACC fibroblasts in showing increased p21 and decreased SRSF3 transcript levels. *P-values<0.05. d. Western blotting experiments show increased p21 protein levels in ACC fibroblasts compared to control fibroblasts, while p53 protein levels are not significantly changed. Relative values for bands normalized to loading control are shown.

Microarray gene expression experiments showed differential expression of 459 genes (p-value<0.05, FDR<5%) between ACC^BMS1(p.R930H)^ fibroblasts and cells obtained from an unaffected unrelated individual (Table S1). Increased expression of p21 (CDKN1A) mRNA in ACC fibroblasts was noticed, which was further confirmed by semiquantitative RT-PCR (Fig. 4a and 4b). The cyclin-dependent kinase inhibitor p21 mediates a G1/S phase arrest in response to cellular stress and other stimuli, and the increased p21 mRNA levels are consistent with the observed G1/S phase transition delay in ACC fibroblasts. Western blotting experiments confirmed increased p21 protein levels in ACC fibroblasts compared to control fibroblasts, whereas total p53 protein levels were not significantly increased (Fig. 4d). There was also no difference in p53^Lys342^ acetylation and no p53^Ser15^ phosphorylation was detected in ACC fibroblasts (data not shown). These findings suggest a role for increased p21 levels in the observed G1/S phase transition defect in ACC fibroblasts. Serine/arginine-rich splicing factor 3 (SRSF3) was found to be downregulated in ACC fibroblasts (Fig. 4a and 4b), which has recently been reported to promote transcription of G1/S phase checkpoint regulators and silencing of SRSF3 caused a G1/S phase arrest (Kurokawa et al., EMBO meeting 2011 abstract).

Overexpression of the mutant BMS1 p.R930H in wild-type fibroblasts resulted in increased transcript levels of p21 and decreased SRSF3 transcript levels, as observed in ACC^BMS1(p.R930H)^ fibroblasts (Fig. 4c).

**Figure 5:**
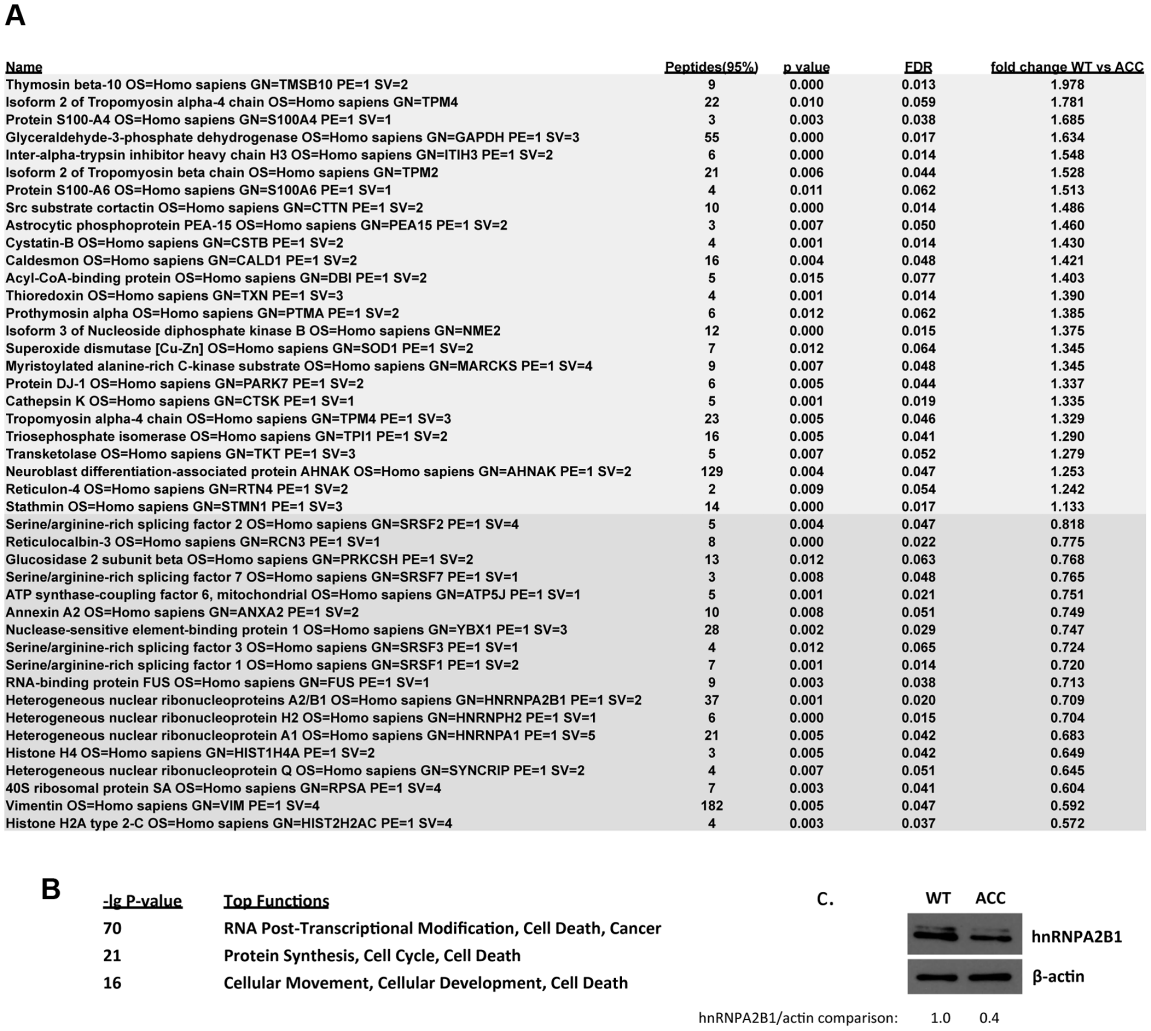
Global proteomic analysis in ACC skin fibroblasts. a. Comparative quantitative proteomic analysis with iTRAQ labeling experiments. Upregulated proteins in ACC fibroblasts are shown in the upper part of the figure and downregulated proteins in the lower part of the figure. The columns show values for the following: Peptides (95%): number of peptides identified with at least 95% confidence; raw p-value; false discovery rate (FDR), fold change of WT vs ACC mean values. b. Network analysis (Ingenuity) identified the most significantly altered networks to include top functions for “RNA-posttranscriptional modification”, “protein synthesis” and “cell cycle” (Fisher exact test –lg p-values indicated). c. Western blotting experiments confirm downregulation of hnRNPA2B1 in ACC fibroblasts compared to control fibroblasts. Relative values for bands normalized to loading control are shown.

Next, a global quantitative comparative proteomic analysis was performed using iTRAQ-labeling and subsequent MS analysis, comparing control versus ACC early passage subconfluent fibroblasts. Using stringent parameters for statistical significance, 25 proteins were found to be consistently upregulated in ACC fibroblasts, whereas 18 were downregulated (Fig. 5a). Downregulated proteins in ACC fibroblasts included several serine/arginine-rich splicing factors (SRSF1, SRSF2, SRSF3, SRSF7), including SRSF3 that was also downregulated in the microarray transcript profiling experiments. Other downregulated proteins included heterogenous nuclear ribonucleoproteins (HNRNPA2B1, HNRNPH2, HNRNPA1). Decreased levels of hnRNPA2B1 in ACC fibroblasts were further confirmed by Western blotting experiments (Fig. 5c). Importantly, hnRNPA2 knockdown has been shown to result in a p53-independent increase of p21 levels and an inhibition of cell proliferation [13], as observed here in ACC^BMS1(p.R930H)^ fibroblasts.

**Figure 6:**
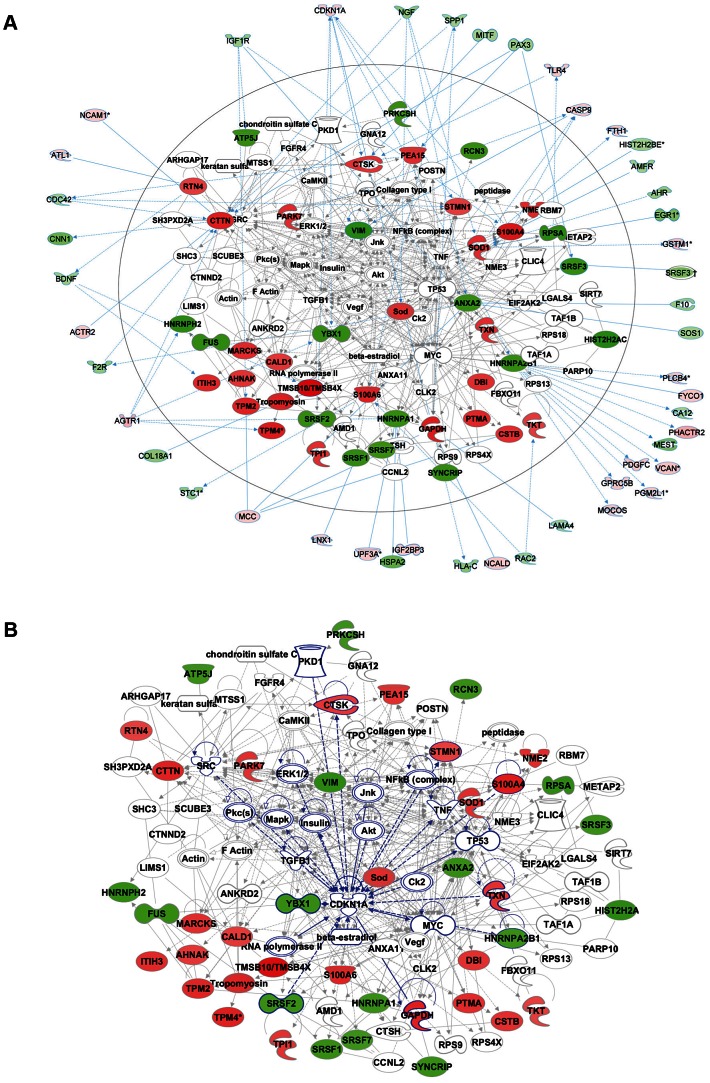
Comparative proteomics and global gene expression profiling reveals a central role of p21 activation in ACC. a. The two top-ranked networks were merged to generate a combined network of the differentially present proteins in ACC fibroblasts (center). Differentially expressed genes identified in the transcript profiling microarray experiments are indicated in the periphery of the proteomic network, and interactions are illustrated by arrows. This analysis revealed the highest number of interactions to include p21 (CDKN1A) and hnRNPA2B1. b. Interaction analysis among p21 (CDKN1A) and the entire merged proteomic network revealed a central role of p21, showing that p21 activation is a central determinant of the ACC phenotype.

Functional enrichment analysis of the proteomic data ranked the category “RNA post-transcriptional modification” as the top-ranked cellular process altered in ACC fibroblasts (Benjamini-Hochberg multiple test corrected p-value 4.72E-06). Network analysis identified the two most significantly altered networks to include top functions for “RNA-posttranscriptional modification” and “cell cycle” (Fisher exact test –lg p-value of 70 and 21 respectively) (Fig. 5b). Thus, the results of the unbiased global proteomic analysis in ACC^BMS1(p.R930H)^ fibroblasts are consistent with a defect in pre-rRNA processing. These two top-ranked networks were merged to generate a combined network of the differentially present proteins in ACC^BMS1(p.R930H)^ fibroblasts (Fig. 6a), which were further analyzed for interactions with the differentially expressed transcripts. This analysis revealed the highest number of interactions to include p21 (CDKN1A) and hnRNPA2B1 (Fig. 6a). Furthermore, interaction analysis among p21 and the entire merged proteomic network revealed a central role of p21, suggesting that p21 activation is a central determinant of the ACC phenotype (Fig. 6b).

## Discussion

Mutations in genes for structural proteins of the ribosome or in other genes involved in ribosome biogenesis or function have been found in rare congenital diseases termed ribosomopathies, including Diamond Blackfan anemia (e.g. RPS19 and RPS24), Shwachman-Diamond syndrome (SBDS), X-linked dyskeratosis congenita (DKC1), cartilage hair hypoplasia (RMRP) and Treacher Collins syndrome (TCOF1, POLR1C and POLR1D) [14], [15], [16], [17], [18]. However, a disease phenotype in these disorders may not always be a direct consequence of ribosomal dysfunction, but may be due to other disease mechanisms. For example, in both dyskeratosis congenita and cartilage hair hypoplasia ribonucleoprotein complexes containing telomerase are involved, likely critical to maintain stem cell function. RMRP, which is mutated in cartilage hair hypoplasia, interacts with TERT (the catalytic subunit of telomerase) and forms a ribonucleoprotein complex that has RNA-dependent RNA polymerase activity [19]. DKC1, mutated in X-linked dyskeratosis congenita, is associated with small nucleolar RNAs, but also with human telomerase RNA (TERC), and it has been suggested that the disease phenotype in dyskeratosis congenita results from a defect in telomere maintenance [20]. Thus, although these two diseases have been classified as ribosomopathies, their pathologies may result from cellular dysfunction that is not due to a ribosome biogenesis defect.

Notably, ribosomopathies manifest with very specific clinical features affecting only few organ systems and often resulting in hematologic and craniofacial abnormalities [21]. These specific phenotypes affect particular cell types, despite the ubiquitous expression of the mutated gene, and illustrate that a mutation in a gene involved in ribosomal function that affects basic cellular pathways can manifest with a selected clinical abnormality. In this context, it is not surprising that despite the ubiquitous expression of BMS1, individuals harboring the BMS1 p.R930H mutation in non-syndromic ACC display only a localized skin morphogenesis defect without further systemic anomalies. A mutation that slows cell proliferation may manifest itself at an anatomic location that requires rapid growth of a tissue compartment, such as the embryonic skin at the vertex area during the rapid expansion of the skull in embryonic development. As such, it is also not surprising that aplasia cutis can be seen in various diverse syndromes in which cell proliferation rate is affected in addition to other basic cellular pathways that also affect other organ systems and lead to multiple congenital anomalies. For example, Adams-Oliver syndrome (AOS) patients have aplasia cutis and multiple additional congenital anomalies, and skin fibroblasts from AOS families with a mutation in a Cdc42/Rac1 regulatory protein (ARHGAP31) showed an enhanced cell migration rate and a reduced cell proliferation rate in vitro, as seen here in ACC^BMS1(p.R930H)^ fibroblasts [3].

Several studies have shown that ribosomal gene mutations can lead to “nucleolar stress” and a G1/S phase cell cycle arrest both via p53-dependent and p53-independent mechanisms that are only partially understood [21], [22]. It has been proposed that the ribosomal stress response, as a consequence of alterations in ribosomal assembly or processing, results in increased free ribosomal proteins that can either inactivate Mdm2, resulting in p53 accumulation and p21-mediated cell cycle arrest, or increase p27 levels that ultimately result in a cell cycle arrest [11]. Consistent with these findings in some ribosomopathies, ACC fibroblasts with the BMS1 p.R930H mutation showed a G1/S phase cell cycle transition defect associated with increased p21-levels that result in a reduced cell proliferation rate.

BMS1 function has been studied mostly in yeast, demonstrating that inducible depletion of BMS1 in yeast results in inhibition of pre-rRNA processing at sites A_0_, A_1_ and A_2_ affecting the small ribosomal subunit processome [7], [8], [9], [10], while little is known about the function of BMS1 in vertebrates. The data presented here are consistent with the findings in yeast and suggest that reduced BMS1 function delays maturation of the 18S rRNA in human cells.

A homozygous mutation in the GTPase domain of BMS1 has recently been reported to result in impaired liver development in zebrafish, while heterozygosity for this mutation does not cause an observable phenotype [23]. This observation suggests a role for BMS1 and other components of the small subunit processome in liver development, which is further supported by the identification of homozygous mutations in the gene cirhin in North American Indian childhood cirrhosis that is required for proper 18S rRNA maturation [24], [25].

The heterozygous BMS1p.R930H mutation in ACC is located in the putative regulatory GAP domain, and therefore is likely to affect BMS1 function to a lesser degree than the homozygous mutation in the GTPase domain of BMS1 in zebrafish. In this context, it is not surprising that patients with autosomal dominant ACC do not display major developmental liver abnormalities. Instead, the data presented here show a developmental localized skin formation defect at a site of rapid expansion of the skin due to a heterozygous mutation in the putative GAP domain of BMS1, which provides the first human mutation for BMS1.

Through which exact mechanisms this mutation causes a p21-mediated G1/S phase arrest and is correlated with the observed downregulation of hnRNPs and SRSFs remains to be determined in future studies. The reported observation that hnRNPA2 knockdown results in a p53-independent increase of p21 levels and an inhibition of cell proliferation [13], similar as observed here in ACC^BMS1(p.R930H)^ fibroblasts, together with the results form the interaction analyses of the proteomic data that show the largest number of interactions to involve p21 and hnRNPA2B1 in ACC fibroblasts, suggest that the reduced protein levels of hnRNPA2B1 likely play a role in ACC pathogenesis.

During embryonic development p21 expression correlates with arrest of cell proliferation and is found in postmitotic cells immediately adjacent to the proliferative compartment, as in the outer embryonic epidermis and particularly in the developing hair follicles [26], [27]. Notably, p21 expression is decreased in terminally differentiated cells and overexpression of p21 inhibits late stages of differentiation of keratinocytes and the stem-cell potential of keratinocyte subpopulations [28], [29]. Thus, the increased p21 levels in ACC may result in an inhibition of cell proliferation as well as an inhibition of terminal differentiation of the outer epidermis during embryonic development, and explain the observed skin morphogenesis defect in ACC. Increased p21 levels have also been linked to increased scar formation, which is consistent with the prominent hypertrophic scar formation in patients with ACC, as was also observed in affected members of this family (Fig. 1a) [30].

In summary, the findings in this study show that the BMS1 p.R930H mutation is associated with a downregulation of hnRNPA2B1 (and other hnRNPs and SRSFs) and a p21 upregulation, leading to a G1/S cell cycle phase transition delay and an inhibition of cell proliferation. The data presented here provide a novel link between BMS1, a p21-mediated cell cycle arrest and skin morphogenesis.

## Materials and Methods

### Patients and samples

A five-generation family was identified with autosomal dominant inheritance of ACC. All participants provided written consent, and the Institutional Review Board of Massachusetts General Hospital approved this study. Genomic DNA was extracted from peripheral blood lymphocytes using the QIAGEN Puregene blood isolation kit (Qiagen). DNA concentrations were determined using the Picogreen assay (Life Technologies).

### Genome-wide SNP and microsatellite genotyping and linkage analysis

Genomic DNA from individuals of this family were prepared, labeled and hybridized to the Affymetrix Genome-Wide Human SNP array 6.0, which features more than 906,000 SNPs. SNP data was analyzed with the Affymetrix Genotyping Console. Merlin version 1.1.2 was used for parametric linkage analysis [31]. Linkage analysis was performed with either 100% or 95% disease penetrance, equal or calculated allele frequencies, and various disease allele frequencies. The frequency of ACC in the population has been estimated to be about 1∶30,000 births. Independently, microsatellite genotyping was performed using informative markers from A&B Biosciences that spanned chromosome 10.

### Targeted capture and massive parallel sequencing

About 10 µg of DNA from one affected individual of this family was used for exome capture using the Agilent Sure select 50 Mb kit. Sequencing was performed on an Illumina HiSeq 2000 sequencer. Reads were mapped to the UCSC hg19 reference human genome. Sequence data was analyzed and variants were filtered using the DNAnexus software package.

### Sequence validation

Bidirectional Sanger sequencing of PCR amplicons from genomic DNA was used to confirm the presence and identity of variants identified via exome sequencing.

### Cell culture and immunocytochemistry

Primary fibroblasts were obtained from a family member with ACC (p.R930H) through a 4 mm abdominal skin biopsy. Control fibroblasts were obtained from a skin biopsy of a matched unrelated individual without ACC (matched for anatomic location, skin phototype, and age). The skin biopsy sample was treated with collagenase I, and subsequently fibroblasts were maintained in culture in DMEM (Invitrogen) with 20% fetal calf serum (Sigma) and antibiotic/antimycotic (Invitrogen).

For cell immunolabeling experiments, fibroblasts were grown on poly-L-lysine coated glass slides (BD Biosciences) and fixed with methanol. Primary antibodies used were as follows: polyclonal rabbit BMS1 (Sigma), monoclonal mouse BMS1 (Santa Cruz), rabbit polyclonal nucleophosmin (Invitrogen), rabbit polyclonal anti-phospho Histone H3 (Ser10) (Millipore). Secondary antibodies used were fluorescently labeled Alexa antibodies from Invitrogen. Nuclei were stained with DAPI. Mouse embryos at E13.5 were fixed in 4% PFA and embedded in OCT and cryosections were used for immunofluorescence experiments. F-actin labeling was performed with phalloidin-Alexa Fluor 488 conjugates (Invitrogen). Microscopy was performed with a Zeiss Axiovert microscope.

### In vitro mutagenesis and expression of wild-type and mutant BMS1 in fibroblasts

In vitro mutagenesis was performed according to the manufacturers protocol (Stratagene). Mutant (c.2789G>A) and wild-type BMS1 cDNA was cloned into the pEGFPN1 (Clontech) expression vector, fusing EGFP at the C-terminus of the expressed cDNA. Primary human wild-type fibroblasts that were used as controls in all experiments (matched for age-, anatomic location and skin phototype) were transfected in triplicate with purified plasmid using XtremeGene HP DNA transfection reagent (Roche) or nucleofection using the Amaxa human dermal fibroblast nucleofector kit with program U-023 (Amaxa). Expression and subcellular localization of the expressed EGFP-tagged protein was evaluated on a Zeiss Axiovert fluorescence microscope. For semiquantitative RT-PCR experiments, transfected fibroblasts (n = 3 independent transfections) were sorted for EGFP 48 hours after transfection using a cell sorter (MoFlo) and RNA was immediately isolated after sorting with Trizol reagent (Invitrogen). Overexpression was confirmed by semiquantitative RT-PCR for BMS1 transcript levels. All experiments were performed in triplicate.

### Cell cycle analysis

Cell cycle analysis with propidium iodide was performed according to standard protocols. Briefly, subconfluent fibroblasts were fixed in 70% ethanol, treated with RNase A and stained with propidium iodide (Sigma) and cell cycle status was determined by FACS analysis and subsequent analysis by FlowJo software (version 9.4.9) using the Watson pragmatic model for determination of cell cycle phases. Experiments were performed in triplicate for ACC and WT cells.

### Cell proliferation assay

The CyQUANT fluorescence-based microplate assay was used for quantitation of cell numbers and used according to the manufacturers protocol (Invitrogen). Binding to cellular nucleic acids was measured by using 485 nm excitation and 530 nm emission filters with a fluorescence microplate reader. The fluorescence emission of the dye-nucleic acid complexes was then correlated linearly with cell numbers from a dilution series of cells that were collected d2–d7 after cell culture of equal numbers of cells. For each time point triplicate experiments were performed.

### In vitro cell migration assay

Cell migration assays were performed with WT and ACC^BMS1(p.R930H)^ primary dermal fibroblasts plated on fibronectin-coated 6 cm^2^ tissue culture dishes (n = 3/group). Cells were serum- and growth-supplement- starved for 12 hours before inducing a linear scratch wound with a 1 ml pipette tip. Cultures were washed twice with PBS, and wound margins were photographed. The same wound margin fields were photographed after 14 hours, and the scratch areas that were not repopulated by fibroblasts were measured with Zeiss Axiovision software. Similar findings were obtained when dishes were coated with collagen.

### Western blotting

Western blotting was performed with 50 µg of total protein lysate from subconfluent early passage ACC and control fibroblasts. Protein concentrations were determined by a standard Bradford assay. NP-40 lysis buffer and complete protease inhibitor cocktail (Roche) was used. NuPage Bis-Tris 4–12% gradient gels were used. Primary antibodies used were: mouse monoclonal anti-p53 (clone DO-1) (Becton Dickson), rabbit polyclonal anti-β-actin (Thermo), rabbit anti-p21 polyclonal antibody (Thermo Fisher) and mouse anti-HNRNPA2B1 (Millipore). HRP-conjugated secondary antibodies were used from GE. β-actin loading control was performed on each blot. Band intensities were quantified using NIH ImageJ software (NIH, version 1.46).

### Pulse-chase labeling of rRNA with L-[*methyl*-^3^H]methionine

To study the kinetics of pre-rRNA processing pulse-chase labeling with L-[*methyl*-^3^H]methionine was performed on early passage subconfluent ACC^BMS1(p.R930H)^ fibroblasts and control fibroblasts as described [32]. Briefly, cells were cultured on 6-well plates and after being cultured for 15 minutes in methionine-free medium, cells were pulsed with L-[*methyl*-^3^H]methionine at a final concentration of 50 µCi/ml for 30 minutes. 10× methionine medium was used for the chase and total RNA was isolated with Trizol (Invitrogen) at 0, 15, 45, 60 and 180 minutes after the end of the pulse. 1 µg of total RNA was loaded on a 1% formaldehyde-agarose gel and after electrophoresis blotted overnight on a nylon membrane (Ambion). Signal intensity was increased with the EN^3^HANCE spray (PerkinElmer Life) and treatment with carbon tetrachloride (Sigma) as described [32]. The nylon membrane was exposed to Kodak BioMax MS high-sensitivity radiography film (Kodak).

### Comparative proteomic analysis with multiplex iTRAQ

Early-passage fibroblasts isolated from an affected individual with ACC from this family with the BMS1 mutation p.R930H were used for comparative proteomic analysis. Fibroblasts, matched for ethnicity and anatomic location, were used as control. From each individual fibroblasts were used as quadruplicate experimental samples in a 8-plex iTRAQ experiment. Whole cell lysates were generated using Pressure Cycling technology. Collected cells were resuspended in RIPA buffer plus Pefablock (Roche complete tabs, 1 tablet per 10 ml) and 1 mM DTT. The cells were then lysed in a Barocyler NEP2320 instrument from Pressure Biosciences, Inc. (South Easton, MA). Following lysis, proteins were recovered by acetone precipitation. Acetone precipitated material that resuspended in milliQ water was used as the soluble protein extract. Protein concentrations were determined using the Bio-Rad Protein Assay with BSA used to generate a standard curve. The 8-plex iTRAQ assay for multiplexed relative quantitation (AB SCIEX, Foster City, CA) was used to determine the protein level differences between control and ACC cells. Briefly, equal amounts of soluble protein from each fibroblast experimental group (100 µg) was dried in a speed vacuum/centrifuge. Following the recommended 8-plex iTRAQ protocol, dried proteins were resuspended in 21 µl of 500 mM TEAB (pH 8.5), 0.1% SDS. Proteins were then reduced with 5 mM TCEP by incubation at 55°C for 1 hour. Reduced disulfide bonds were then blocked by adding a final concentration of 8 mM MMTS and incubating at room temperature for 10 min. Peptides were generated in each sample by overnight digestion with trypsin (AB SCIEX) added at a ratio of 1∶10. Samples were then labeled with the 8-plex iTRAQ reagent. Confirmation of efficient label attachment was confirmed by MS/MS in an AB SCIEX 4800 MALDI-TOF/TOF before samples were pooled into one.

Half of the sample (∼400 µg) was processed using standard MuDPIT methodology to maximize protein identification. First dimension strong cation exchange (SCX) separation was performed on an Agilent 1100/1200 HPLC with a POROS HS/20 column (4.6×100 mm, AB SCIEX). 40 fractions covering the eluted peptides were then individually run for second dimension reverse phase separation on a Dionex Ultimate Plus nanoLC with a C18 Acclaim PepMap100 column (75 µm×15 cm, 3 µm beads). Approximately 500 spots were printed per fraction to 4800 MALDI plates (5 SCX fractions per plate) with CHCA (5.0 mg/ml) mixed in by the ProBot in a mixing Tee immediately before printing. MS and MS/MS on the peptides were performed in the AB SCIEX 4800 mass spectrometer.

### Proteomic data analysis

Relative abundance quantitation and peptide and protein identification were performed using ProteinPilot software 3.0 (Applied Biosystems). The Swiss-Prot Homo sapiens protein database (Swiss-Prot/UniProt) was used for all searches. The data were normalized for loading error by bias analysis using ProteinPilot.

The high confidence proteins are defined as those with (a) >90% confidence as determined by ProteinPilot (ProtScore ≥1.0) and (b) two distinct peptides with different iTRAQ spectra identified with ≥95% confidence. These high confidence proteins were identified using Perl and R scripts. The differentially present proteins were identified by comparing the relative weighted average iTRAQ values of proteins in the control and ACC groups. The significance of differential levels of protein iTRAQ ratios was determined using the multiple test corrected (FDR) P-value. Proteins that had a iTRAQ ratio >1.2 or <0.80 (calculated on basis of variance in iTRAQ values), a raw P-value of <0.05 and a FDR<10% between control and ACC groups were considered differentially present.

Interactive networks and pathways were analyzed using the Ingenuity Pathways Analysis package (IPA 4.0) (http://www.ingenuity.com/). It calculates the P-value for each network, and function according to the fit of user's data to IPA database. It displays the results as Fisher's exact test -lg P-value indicating the likelihood of a gene to be found in a network or function by random chance.

### Microarray transcript analyses and semiquantitative RT-PCR

Early-passage fibroblasts isolated from an affected individual with aplasia cutis from this family with the BMS1 mutation p.R930H and control fibroblasts, matched for ethnicity and anatomic location (but not for gender), were each used as triplicate experimental samples in gene expression analysis experiments (which were also used in the comparative proteomic analysis).

Total RNA was isolated from fibroblasts using the RNEasy Plus kit (Qiagen), and treated with DNAse. Semiquantitative RT-PCR was performed using the total RNA. cDNA was generated using the Transcriptor First Strand cDNA synthesis kit with random hexamer primers (Roche). Semiquantitative RT-PCR was performed using the LightCycler 480 DNA SYBR-Green I Master kit on a LightCycler 480 (Roche Applied Science). The following primers were used: p21 (up: GCAGACCAGCATGACAGATTT; dw: GGATTAGGGCTTCCTCTTGGA). Pretested SRSF3 and BMS1 qPCR primers were used from SABiosciences (Qiagen) and results were normalized to 36B4 transcript levels. Reactions were performed in triplicate (n = 3) and also three times for each sample.

Human Genome U133 Plus 2.0 Affymetrix microarrays were used for global gene expression profiling experiments. Obtained raw data was analyzed using the dCHIP software (https://sites.google.com/site/dchipsoft/). Differential expression was determined significant with dCHIP with a P-value<0.05 and a false discovery rate (FDR) <5% among the control (WT) and ACC groups (samples in triplicate for each group; corrected for gender).

### Inducible knockdown of BMS1

To achieve a stable partial inducible knockdown of BMS1 a puromycin-selectable TRIPZ doxycycline-inducible lentiviral shRNA system was used, which allows for the detection of shRNA-mediated gene silencing by TurboRFP expression (Open Biosystems). TRIPZ BMS1 shRNA clone V3THS_361658 was used to transfect wild-type fibroblasts and cells were selected by puromycin treatment. Fluorescence microscopy confirmed that after puromycin treatment all remaining cells were expressing TurboRFP. After 48 hours treatment with 1 µg/ml doxycycline RNA was isolated from these stably transfected cells to obtain cDNA. Semiquantitative RT-PCR for BMS1 confirmed a ∼50–60% knockdown of BMS1 transcript levels and cDNA was used for semiquantitative RT-PCR of p21 and SRSF3 transcript levels. As controls these cells were used in the absence of doxycycline treatment. From each group experiments were performed in triplicate. Total RNA was used for Northern Blot analyses.

### Northern blotting

Northern blotting was performed as described previously [32]. From each sample 2 µg total RNA was loaded on a 1.2% formaldehyde-agarose gel and blotted on a positively charged Nylon membrane (BrightStar-Plus, Ambion). Radioactive probes labeled with [γ-^32^P]ATP for ITS-1, ETS-1 and ITS-2 were used as described previously [33]. Band intensities were quantified using NIH ImageJ software (NIH, version 1.46).

## Supporting Information

Figure S1 a. Transient transfection of EGFP-tagged full-length mutant (c.2789G>A) and wild-type BMS1 (b.) in control fibroblasts shows nucleolar localization. Thus, the mutation does not affect subcellular localization of BMS1. c.–h. Immunofluorescence labeling of subconfluent ACC and control fibroblasts. c. BMS1 maintains its nucleolar localization (green, N-term) and co-localizes with nucleophosmin (red) as seen in control cells (d. WT). N-term indicates labeling with the polyclonal anti-BMS1 antibody recognizing the N-terminus of BMS1. E–f. Labeling of the C-terminal (C-term, red) and N-terminal domain of BMS1 (N-term, green) shows nucleolar co-localization in ACC (e.) and control fibroblasts (f.). C-term indicates labeling with the monoclonal anti-BMS1 antibody that recognizes the C-terminus of BMS1. g. ACC and control fibroblasts (h.) maintain their ability to proliferate and show staining for the proliferation marker phospho-Histone 3 (Ser10) (pH3b, green). BMS1 expression in red (C-term). i.–j. Phalloidin staining shows no major difference of the actin cytoskeleton in ACC and control cells. Nuclei are labeled with DAPI. All images are acquired with a 20× objective. k. Semiquantitative RT-PCR shows no significant difference of BMS1 expression in ACC and control cells (BMS1 transcript levels normalized to 36B4 transcript levels; P-value>0.05).
(PDF)

Figure S2 Northern blot analysis of pre-rRNA processing using radioactive ETS-1 and ITS-2 probes. RNA from ACC fibroblasts was used carrying the BMS1 p.R930H mutation, as well as from control fibroblasts (WT). RNA from fibroblasts stably transfected with an inducible BMS1 shRNA vector was used, after shRNA-mediated knockdown of BMS1 transcripts was induced by doxycycline (doxy) treatment. Ethidiumbromide stained gels prior to blotting (bottom) confirm equal loading of RNA. Quantitation of band intensity ratios expressed as relative values (fold-change compared to WT or untreated cells). The 12S bands shown for the ITS-2 Northern blot in WT and ACC cells were exposed longer to reveal discernable bands.
(PDF)

Table S1 Microarray expression analysis data of subconfluent early-passage fibroblasts derived from an affected individual with ACC (BMS1p.R930H) and a matched healthy control individual. Transcripts that are at least 1.2-fold differentially expressed are indicated (P-value<0.05).
(PDF)
