# Supplementary figures and images for: BMS1 Is Mutated in Aplasia Cutis Congenita
Source: PLoS Genet. 2013 Jun 13;9(6):e1003573. doi: 10.1371/journal.pgen.1003573 (PMC3681727; doi:10.1371/journal.pgen.1003573)

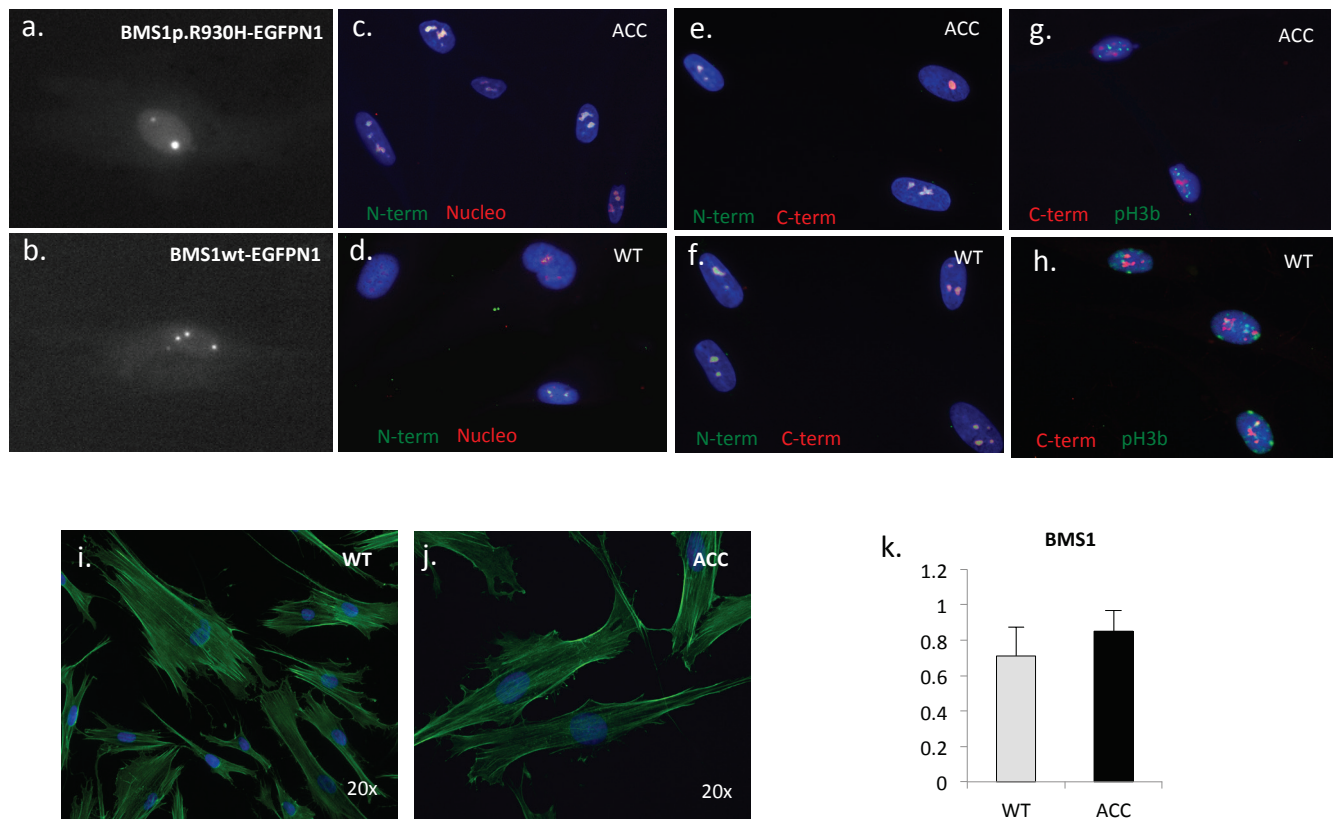

Supplemental Figure 1

Supplement: Figure S1 — a. Transient transfection of EGFP-tagged full-length mutant (c.2789G>A) and wild-type BMS1 (b.) in control fibroblasts shows nucleolar localization. Thus, the mutation does not affect subcellular localization of BMS1. c.–h. Immunofluorescence labeling of subconfluent ACC and control fibroblasts. c. BMS1 maintains its nucleolar localization (green, N-term) and co-localizes with nucleophosmin (red) as seen in control cells (d. WT). N-term indicates labeling with the polyclonal anti-BMS1 antibody recognizing the N-terminus of BMS1. E–f. Labeling of the C-terminal (C-term, red) and N-terminal domain of BMS1 (N-term, green) shows nucleolar co-localization in ACC (e.) and control fibroblasts (f.). C-term indicates labeling with the monoclonal anti-BMS1 antibody that recognizes the C-terminus of BMS1. g. ACC and control fibroblasts (h.) maintain their ability to proliferate and show staining for the proliferation marker phospho-Histone 3 (Ser10) (pH3b, green). BMS1 expression in red (C-term). i.–j. Phalloidin staining shows no major difference of the actin cytoskeleton in ACC and control cells. Nuclei are labeled with DAPI. All images are acquired with a 20× objective. k. Semiquantitative RT-PCR shows no significant difference of BMS1 expression in ACC and control cells (BMS1 transcript levels normalized to 36B4 transcript levels; P-value>0.05). (PDF) [file pgen.1003573.s001.pdf]

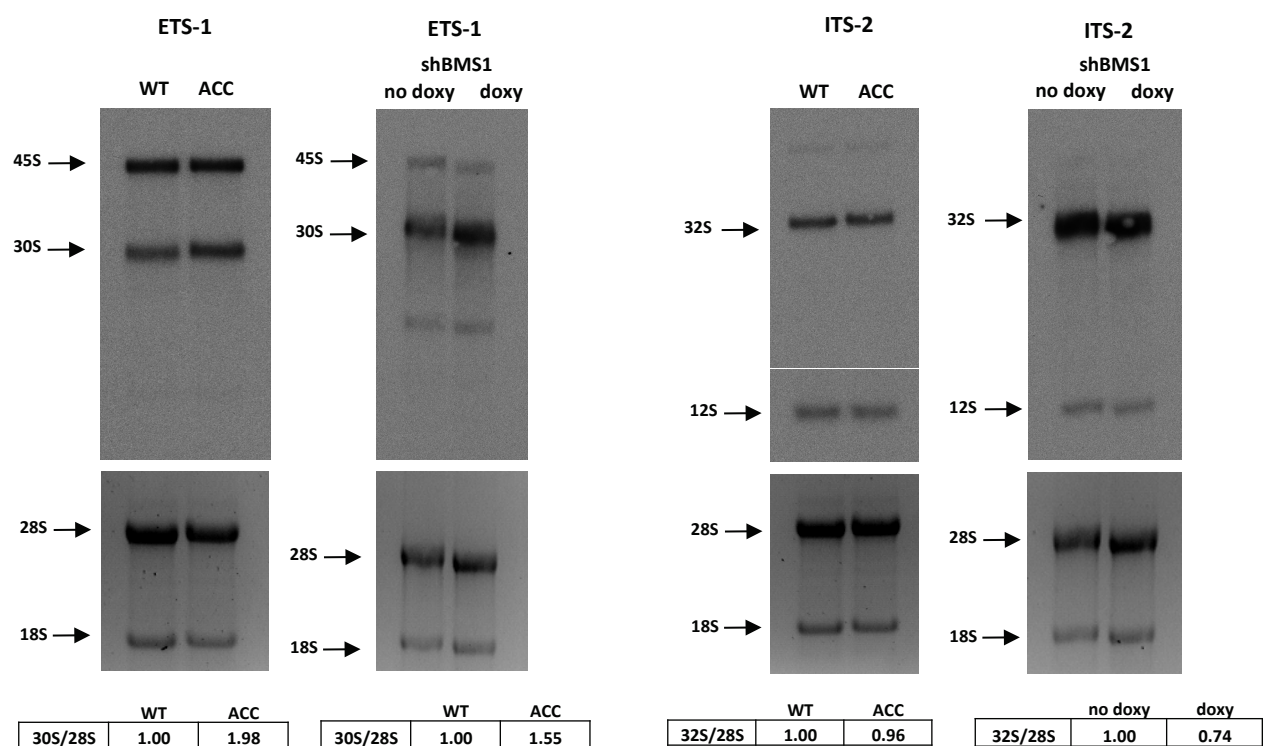

Supplemental Figure 2

Supplement: Figure S2 — Northern blot analysis of pre-rRNA processing using radioactive ETS-1 and ITS-2 probes. RNA from ACC fibroblasts was used carrying the BMS1 p.R930H mutation, as well as from control fibroblasts (WT). RNA from fibroblasts stably transfected with an inducible BMS1 shRNA vector was used, after shRNA-mediated knockdown of BMS1 transcripts was induced by doxycycline (doxy) treatment. Ethidiumbromide stained gels prior to blotting (bottom) confirm equal loading of RNA. Quantitation of band intensity ratios expressed as relative values (fold-change compared to WT or untreated cells). The 12S bands shown for the ITS-2 Northern blot in WT and ACC cells were exposed longer to reveal discernable bands. (PDF) [file pgen.1003573.s002.pdf]
