# Supplementary material for: BMS1 Is Mutated in Aplasia Cutis Congenita
Source: PLoS Genet. 2013 Jun 13;9(6):e1003573. doi: 10.1371/journal.pgen.1003573 (PMC3681727; doi:10.1371/journal.pgen.1003573)
Supplement: Table S1 — Microarray expression analysis data of subconfluent early-passage fibroblasts derived from an affected individual with ACC (BMS1p.R930H) and a matched healthy control individual. Transcripts that are at least 1.2-fold differentially expressed are indicated (P-value<0.05). (PDF) [file pgen.1003573.s003.pdf]

gene	EntrezGene	WT-1	WT-2	WT-3	ACC-1	ACC-2	ACC-3	fold change	P value
ES cell associated transcript 8	91646	55.55	49.08	73.15	3215.4	4201.35	3818.96	63.21	0.00605
similar to Tektin-3 /// hypothetical protein LO	389830 /// 642398	70.54	86.23	98.21	1162.25	1369.02	1484.63	15.94	0.005301
keratin, hair, acidic, 4	3885	238.19	249.77	284.67	2259.44	3607.06	2795.2	11.24	0.021824
similar to Tektin-3 /// similar to hypothetical p	389830 /// 389833	109.69	102.19	94.06	823.66	994.29	1011.85	9.29	0.00193
similar to Tektin-3 /// similar to hypothetical p	389830 /// 389833	114.94	117.51	112.12	844.85	1010.04	1081.49	8.54	0.00415
pregnancy specific beta-1-glycoprotein 7	5676	716.17	195.49	247.58	3006.1	1633.28	1641.98	5.68	0.048266
glutathione S-transferase theta 2	2953	121.22	143.31	120.35	643.02	729.16	666.52	5.31	0.000765
myxovirus (influenza virus) resistance 1, inte	4599	68.22	67.8	72.73	449.76	322.13	291.77	5.1	0.025725
hypothetical LOC654433	654433	93.28	93.23	94.34	417	474.49	420.25	4.67	0.000632
keratin, hair, acidic, 3A	3883	111.11	129.72	125.21	444.51	581.05	496.64	4.19	0.00566
Phosphatase and actin regulator 2	9749	664.52	563.25	507.83	1881.19	1777.22	2808.97	3.73	0.038475
type 1 tumor necrosis factor receptor sheddi	51752	25.04	52.84	59.41	142.18	192.97	171.65	3.7	0.004824
angiotensin II receptor, type 1	185	239.04	251.71	291.39	654.94	1079.38	1063.13	3.61	0.038996
beta 1,3-galactosyltransferase-like	145173	217.12	248.22	274.46	815.24	922.5	924.73	3.6	0.000959
B cell RAG associated protein	51363	133.51	128.79	167.88	601.21	482.66	430.27	3.5	0.014365
churchill domain containing 1	91612	851.62	837.87	868.83	2639.07	2909.05	2722.48	3.24	0.000986
Holocarboxylase synthetase (biotin-(proprio	3141	46.78	50.41	45.26	168.21	144.12	145.54	3.19	0.002204
progestin and adipoQ receptor family memb	54852	202.88	139.95	163.35	481.66	510.62	490.2	2.92	0.000522
progestin and adipoQ receptor family memb	54852	364.99	256.6	309.03	936.11	856.12	862.46	2.85	0.000196
hypothetical protein LOC63929	63929	116.04	116.15	111.25	340.78	305.91	326.78	2.83	0.000378
ST8 alpha-N-acetyl-neuraminide alpha-2,8-s	8128	59.4	60.93	82.1	131.02	224.16	191.07	2.77	0.043205
dickkopf homolog 2 (Xenopus laevis)	27123	59.77	57.96	70.38	151.25	188.78	172.73	2.73	0.00423
hypothetical protein FLJ10781	55228	147.12	108.6	113.04	400.74	311.92	291.5	2.72	0.014368
glutathione S-transferase M2 (muscle)	2946	514.95	709.14	642.72	1557.28	1579.3	1692.33	2.6	0.000549
Elongation factor, RNA polymerase II, 2	22936	143.72	144.13	86.86	257.24	324.61	373.58	2.57	0.015206
hypothetical protein LOC644246 /// hypothet	644246 /// 649063	192.68	237.39	246.07	570	546.7	585.64	2.52	0.000496
glutathione S-transferase M1	2944	505.8	648.79	640.46	1475.4	1510.88	1549.24	2.51	0.000633
lectin, galactoside-binding, soluble, 8 (galec	3964	144.88	98.85	98.72	297.1	268.65	283.17	2.47	0.00181
cholinergic receptor, nicotinic, alpha 10	57053	124.81	122.23	114.92	253.18	387.33	262.33	2.47	0.046359
hypothetical protein LOC130576	130576	283.08	411.89	347.35	809.21	900.4	866.81	2.47	0.000726
prostate collagen triple helix	542767	85.07	93.08	95.47	215.21	232.86	217.65	2.43	0.000763
Similar to FRAS1-related extracellular matri	387904	67.77	75.46	72.95	208.35	158.74	155.62	2.43	0.022485
Ras interacting protein 1	54922	152.73	147.86	154.81	337.84	383.91	346.6	2.35	0.002533
glutathione S-transferase M1	2944	480.24	611.74	563.53	1314.98	1197.95	1302.75	2.3	0.000259
cystin 1 /// similar to cystin 1	192668 /// 649824	79.47	122.45	124.25	185.75	260.72	302.48	2.3	0.040342
interleukin 1 receptor accessory protein	3556	283.12	265.28	292.4	691.34	609.83	590.61	2.24	0.00331
Paired box gene 8	7849	166.36	162.36	155.53	346.55	385.52	349.89	2.24	0.001083
hypothetical LOC653140	653140	83.84	92.45	93.51	219.17	176.45	211.18	2.24	0.00439
sushi domain containing 5	26032	196.35	422.33	497.78	631.65	963.27	868.56	2.21	0.029752
chromosome 6 open reading frame 105	84830	288.89	196.8	229.12	483.87	625.47	467.41	2.2	0.016481
CDNA clone IMAGE:30721737		157.65	123.73	98.61	344.62	250.2	247.88	2.2	0.024324
Guanylate binding protein 5	115362	181.78	169.37	210.12	379.05	425.6	422.11	2.19	0.002063
epithelial stromal interaction 1 (breast)	94240	226.93	202.49	194.81	549.85	409.52	400.47	2.18	0.029081
hypothetical gene supported by AK075484;	339804	487.99	629.25	645.22	1192.08	1285.24	1353.04	2.17	0.00071
endomucin	51705	73.97	111.66	127.29	178.96	240.36	257.2	2.14	0.024701
zinc finger protein 77	58492	208.89	233.95	218.25	396.87	529.09	480.39	2.14	0.020549

ADAM metallopeptidase domain 33	80332	319.62	276.09	279.25	680.95	509.04	686.68	2.13	0.022749
hypothetical protein MGC52057	130574	249.32	202.52	216.01	427.12	488.46	466.95	2.1	0.001598
Aldo-keto reductase family 1, member C2 (d	1646	139.33	134.42	135.08	268.77	307.54	270.41	2.09	0.00706
cysteine dioxygenase, type I	1036	212.8	209.09	245.07	382.2	484.03	502.35	2.08	0.015847
carboxypeptidase E	1363	445.28	611.36	680.08	1177.2	1149.6	1273.46	2.07	0.004187
ribosomal protein S23	6228	291.62	179.84	236.81	568.33	374.86	514.5	2.05	0.030784
hypothetical protein MGC52057	130574	396.12	301.19	335.75	721.33	707.88	671.71	2.04	0.000917
molybdenum cofactor sulfurase	55034	288.78	375.74	389.15	609.84	769.19	755.75	2.03	0.00728
hypothetical protein LOC286434	286434	298.2	268.41	266.83	490.35	555.34	592.88	1.97	0.006158
gb:BF002195 /DB_XREF=gi:10702470 /DB_XREF=7h23h07.x		194.74	288.86	308.14	563.13	448.44	553.49	1.97	0.009035
low density lipoprotein receptor-related prote	84918	2718.21	2339.09	2599.18	5798.74	4460.3	4617.47	1.94	0.022967
CDC42 small effector 2	56990	249.95	256.1	258.69	399.49	566.44	514.97	1.94	0.039286
hypothetical gene supported by BC041875;	400642	199.6	244.08	241.25	408.11	463.15	434.61	1.91	0.001322
Full-length cDNA clone CS0DF025YA01 of Fetal brain of Homo		342.88	335.55	382.27	640.55	716.62	675.53	1.91	0.001877
ADAM metallopeptidase domain 12 (meltrin	8038	125.02	108.11	129.88	255.22	226.16	219.07	1.91	0.003154
golgi autoantigen, golgin subfamily a, 8B	440270	185.28	208	191.71	326.87	371.45	401.21	1.89	0.006801
transmembrane protein 16C	63982	197.83	147.27	161.45	390	272.47	299.83	1.88	0.036109
ribonuclease P 25kDa subunit	54913	508.33	344.94	390.08	789.02	810.19	721.03	1.85	0.005945
FLJ20160 protein	54842	446.6	348.92	372.75	752.21	711.77	695.68	1.84	0.001689
cadherin 13, H-cadherin (heart)	1012	518.66	959.92	904.42	1121.55	1524.9	1705.57	1.83	0.044931
hypothetical protein KIAA1833	377711	444.6	283.85	308.55	755.01	583.85	549.23	1.83	0.02659
similar to FRG1 protein (FSHD region gene	642236	276.06	282.61	315.08	441.68	588.82	578.99	1.83	0.028274
phospholipase C, beta 4	5332	910.39	982.85	1183.4	1567.21	2025	2015.44	1.82	0.017133
proliferation-inducing protein 38	55068	202.94	253.96	254.81	337.74	487.42	461.75	1.81	0.041791
Transcribed locus		216.41	236.32	279.99	401.19	473.14	452.82	1.81	0.003618
COBL-like 1	22837	895.48	759.62	909.33	1502.15	1538.46	1557.9	1.8	0.000813
butyrophilin, subfamily 3, member A2	11118	414.69	419.58	395.06	819.18	657.36	719.26	1.79	0.016862
hypothetical protein FLJ21901	79675	996.43	773.67	870.55	1500.78	1661.2	1562.96	1.79	0.00136
CDNA clone IMAGE:3960708		604.84	582.83	580.03	1159.7	997.22	1007.84	1.79	0.006381
phospholipase C, beta 4	5332	1003.77	981.28	987.6	1769.05	1475.58	2047.86	1.78	0.040406
Zic family member 1 (odd-paired homolog, D	7545	186.41	296.63	330.54	401.47	508.64	539.2	1.78	0.02744
sphingomyelin phosphodiesterase 1, acid ly	6609	1513.29	1623.8	1709.62	2813.89	2877.96	2912.12	1.78	0.000118
UDP-N-acetyl-alpha-D-galactosamine:polyp	11227	424.96	485.75	538.63	1011.8	733.8	847.03	1.78	0.026154
GDNF family receptor alpha 1	2674	139.7	153.03	177.99	232.5	328.35	271.15	1.77	0.039479
chromosome 7 open reading frame 10	79783	290.8	413.76	363.98	641.67	614.21	636.8	1.77	0.01206
mutated in colorectal cancers	4163	467.42	329.75	365.42	837.28	627.28	588.04	1.77	0.041239
spermatogenesis associated 18 homolog (ra	132671	424.11	620.95	675.57	1110.92	968.21	964.77	1.76	0.01375
chromosome 11 open reading frame 54	28970	441.57	500.42	487.58	916.82	789.74	788.06	1.74	0.005697
ligand of numb-protein X 1	84708	355.16	267.4	286.61	536.84	502.77	524.72	1.74	0.007265
Full-length cDNA clone CS0DF027YP13 of Fetal brain of Homo		405.65	400.55	483.89	617.96	792.66	798.12	1.71	0.025936
MLF1 interacting protein	79682	3056.34	2562	2570.34	4492.25	4869.91	4548.41	1.71	0.001245
aldehyde dehydrogenase 1 family, member A	220	4371.68	3864.25	4386.56	7797.14	6699.99	6951.97	1.7	0.004941
DKFZP564C152 protein	26120	307.85	387.96	377.31	532.81	661.13	630.89	1.7	0.009714
transporter 2, ATP-binding cassette, sub-fan	6891	349.14	312.89	332.75	538.53	591.75	548.18	1.7	0.003158
MRNA; cDNA DKFZp762I0915 (from clone DKFZp762I0915)		163.82	171.52	198.32	270.43	340.19	305.67	1.7	0.015537
MLF1 interacting protein	79682	282.15	231.04	225.94	371.66	447.54	411.75	1.67	0.007021
Hypothetical protein LOC642775	642775	723.91	623.99	710.58	1182.28	1117.57	1089.53	1.66	0.000795

Chromosome 21 open reading frame 113	378825	150.27	156.47	145.17	260.28	247.52	244.75	1.66	0.004301
Transcribed locus		223.12	135.55	156.23	262.72	317.73	272.77	1.65	0.031952
Hypothetical protein LOC143381	143381	253.1	304.42	352.35	454.24	532.43	523.51	1.65	0.009729
copine I	8904	1027.75	978.03	961.79	1805.8	1539.19	1557.84	1.64	0.009106
neural cell adhesion molecule 1	4684	317.14	366.16	359.01	502.45	600.34	611.07	1.64	0.013894
fibronectin type III domain containing 1	84624	335.98	303.06	295.43	435.25	550.12	553.21	1.64	0.027133
transmembrane protein 17	200728	285.05	331.88	308.97	534.63	480.53	516.06	1.64	0.002253
dihydropyrimidinase-like 4	10570	201.73	217.31	244.05	321.06	407.38	352.4	1.63	0.022118
hypothetical protein MGC52057	130574	233.52	190.18	215.77	320.9	376.92	340.54	1.63	0.007179
NGFRAP1-like 1	340542	197.15	222.06	210.15	330.26	332.95	358.52	1.62	0.009676
programmed cell death 6 /// hypothetical pro	10016 /// 650280 /	268.73	231.81	244.68	392.43	406.75	405.35	1.62	0.000862
spastic paraplegia 3A (autosomal dominant)	51062	351.08	366.65	361.83	577.35	547.73	616.39	1.61	0.003281
tumor necrosis factor (ligand) superfamily, m	8742	360.72	310.53	336.77	626.04	500.55	494.6	1.6	0.031221
docking protein 5	55816	2409.6	1900.25	2125.47	4001.35	3188.24	3126.69	1.6	0.027122
hypothetical gene supported by AK128780 /.	388312 /// 399844	244.77	321.59	294.47	400.18	507.89	486.57	1.6	0.020061
aldo-keto reductase family 1, member C1 (d	1645	9831.87	7086.82	6497.3	14521.43	11506.52	11328.23	1.59	0.033715
phosphoglucomutase 2-like 1	283209	953.75	814.18	771.58	1393.38	1261.4	1386.44	1.59	0.002564
prostaglandin F2 receptor negative regulato	5738	374.57	386.17	390.87	688.66	573.26	564.16	1.58	0.020242
crystallin, zeta (quinone reductase)	1429	1888.54	2001.11	2113.88	2633.98	3408.61	3349.63	1.57	0.037547
3'-phosphoadenosine 5'-phosphosulfate syn	9060	6000.06	6492.67	7130.32	10926.4	10028.22	10036.85	1.57	0.001611
periostin, osteoblast specific factor	10631	14796.2	15365.66	15926.17	25941.34	23531.32	22956.16	1.57	0.004476
poliovirus receptor	5817	691.85	605.82	630.4	984.98	1054.37	997.56	1.57	0.001189
transmembrane 9 superfamily member 3	56889	5204.53	4282.73	4636.13	7575.15	7215.15	7393.39	1.57	0.00433
similar to RIKEN cDNA 1110018M03	387758	393.03	451.94	443.53	628.03	696.13	711.14	1.57	0.005316
Neural cell adhesion molecule 1	4684	360.25	544.1	558.67	642.69	815.27	845.82	1.57	0.042558
aldo-keto reductase family 1, member C1 (d	1645	12800.43	9670.58	9130.71	18247.55	16046.6	14999.6	1.56	0.018506
aldo-keto reductase family 1, member C2 (d	1646	10666.9	7993.42	7544.51	15597.7	12939.5	12488.8	1.56	0.023857
gb:BF437011 /DB_XREF=gi:11449329 /DB_XREF=7p61e06.x		604.1	503.24	538.85	898.4	875.78	792.5	1.56	0.00343
chromosome 11 open reading frame 54	28970	281.52	326.01	274.65	538.51	384.11	458.12	1.56	0.049211
vacuolar protein sorting 26 homolog A (yeas	9559	7433.31	5844.86	6172.09	11837.64	9545.05	8972.64	1.55	0.034657
1-aminocyclopropane-1-carboxylate synthas	84680	165.08	208.83	196.6	291.32	284.75	305.23	1.55	0.011066
tripartite motif-containing 5	85363	414.36	456.45	458.8	634.92	691.59	724.58	1.54	0.004436
phosphogluconate dehydrogenase /// phosp	5226	1315.05	1644.15	1549.64	2299.52	2013.08	2573.57	1.53	0.020334
exostoses (multiple) 2	2132	1765.18	2076.81	2301.26	2918.43	3405.48	3103.52	1.53	0.009729
insulin-like growth factor 2 mRNA binding pr	10643	339.46	238.85	292.93	434	489.9	407.46	1.53	0.018722
butyrophilin, subfamily 3, member A2	11118	252.53	240.74	267.73	414.02	391.86	361.97	1.53	0.00471
N-acylsphingosine amidohydrolase (acid cer	427	1082.73	1391.44	1472.35	1993.79	2070.17	2001.57	1.53	0.021914
TBC1 domain family, member 2	55357	488.24	508.76	541.48	729.04	857.45	768.64	1.52	0.011402
Hypothetical protein LOC642351	642351	225.85	229.13	232.12	315.71	372.33	355.2	1.52	0.017348
Transcribed locus		234.52	216.84	248.39	355.77	336.45	371.03	1.52	0.002142
actin, alpha, cardiac muscle	70	8377.41	9334.73	10301.08	14273.07	13638.32	14422.93	1.51	0.006015
HIV-1 Tat interactive protein 2, 30kDa	10553	2564.62	2434.28	2490.71	4112.21	3675.68	3554.57	1.51	0.011348
platelet derived growth factor C	56034	891.07	735.96	728.12	1267.19	1131.07	1149.89	1.51	0.005501
G protein-coupled receptor, family C, group	51704	266.41	254.34	244.58	435.52	338.51	382.7	1.51	0.035088
iroquois homeobox protein 1	79192	896.67	890.07	895.07	1364.17	1344.38	1334.12	1.51	0.000095
proteoglycan 1, secretory granule	5552	491.19	555.11	577.76	841.3	772.86	828.74	1.5	0.003355
butyrophilin, subfamily 3, member A3 /// buty	10384 /// 11118	675.96	683	693.96	1132.3	998.42	954.24	1.5	0.017933

laminin, alpha 2 (merosin, congenital muscu	3908	685.74	830.43	872.1	1148.26	1236.7	1192.02	1.5	0.00857
GUF1 GTPase homolog (S. cerevisiae)	60558	816.54	619.21	771.54	1038.24	1187.39	1075.66	1.5	0.010527
Zinc finger, DHHC-type containing 3	51304	207.8	210.57	208.17	278.32	351.17	314.52	1.5	0.034175
hypothetical protein	22998	225.78	253.66	248.6	385.25	306.12	393.92	1.49	0.038896
Transcribed locus		225.5	275.09	304.2	352.16	432.14	424.95	1.49	0.029549
CWF19-like 1, cell cycle control (S. pombe)	55280	874.47	782.52	876.41	1082.33	1439.04	1271.07	1.49	0.049873
vacuolar protein sorting 41 (yeast)	27072	310.7	386.22	434.14	607.49	546.4	551.64	1.49	0.02406
Transcribed locus, moderately similar to NP_055301.1 neurona		266.52	210.52	226.77	341.42	366.16	351.68	1.49	0.011779
zinc finger protein 575	284346	315.59	287.36	354.09	497.4	475.83	456.1	1.49	0.005809
signal-regulatory protein alpha	140885	1628.82	1886.59	2036.27	2481.93	2898.8	2854.26	1.48	0.008867
ninjurin 1	4814	351.51	394.88	390.09	554.29	548.72	584.19	1.48	0.002091
zinc fingers and homeoboxes 2	22882	490.13	485.01	544.67	745.61	775.59	725.65	1.48	0.001168
golgi autoantigen, golgin subfamily a, 8G ///	283768 /// 388080	381.02	392.03	389.94	509.69	600.19	625.93	1.48	0.026064
nudix (nucleoside diphosphate linked moiety	55270	2276.76	1890.61	1886.4	2940.88	3009.4	2995.16	1.48	0.011933
toll-like receptor 4 /// toll-like receptor 4	7099	220.82	258.02	288.75	395.42	344.25	394.79	1.48	0.011079
Archaemetzincins-2	51321	363.94	347.64	372.55	564.21	527.74	521.67	1.48	0.00279
CDNA FLJ46881 fis, clone UTERU3015647, moderately simila		943.65	750.1	742.47	1312.37	1210.48	1116.15	1.48	0.013285
phosphoglucomutase 2-like 1	283209	358.05	265.54	330.83	464.08	521.57	428.62	1.48	0.019012
hypothetical LOC401093	401093	246.94	220.59	248.26	351.98	349.2	352.01	1.48	0.002819
cyclin-dependent kinase inhibitor 1A (p21, C	1026	7136.11	7395.06	7263.86	11217.1	10913.18	9870.4	1.47	0.010392
glycine receptor, beta	2743	571.3	423.97	438.86	638.69	776.76	689.92	1.47	0.024238
transient receptor potential cation channel, s	7223	232.79	241	259	335.16	348.65	395.12	1.47	0.015232
transcription elongation factor A (SII), 3	6920	211.35	272.48	263.02	428.65	326.62	352.29	1.47	0.040885
phosphoinositide-3-kinase, catalytic, delta p	5293	781.74	658.22	692.52	912.93	1113.88	1074.33	1.46	0.019375
ferritin, heavy polypeptide 1	2495	4954.6	6960.84	6958.89	9418.91	9104.29	9090.16	1.46	0.04717
nexilin (F actin binding protein)	91624	918.61	890.46	963.24	1224.96	1390.86	1434.58	1.46	0.013573
gb:BF059209 /DB_XREF=gi:10813105 /DB_XREF=7k56b09.x		255.83	301.8	335.7	371.91	488.75	450.58	1.46	0.040463
laminin, alpha 2 (merosin, congenital muscu	3908	459.67	534.58	538.86	739.56	699.92	774.56	1.45	0.004946
cat eye syndrome chromosome region, canc	27440	1371.2	1372.55	1382.56	1912.69	2133.09	1954.27	1.45	0.007635
transmembrane protein 80	283232	720.24	779.76	899.22	1051.6	1231.1	1201.16	1.45	0.012568
G protein-coupled receptor 177	79971	1265.91	884.67	951.27	1576.14	1525.26	1383.35	1.45	0.040695
CDNA FLJ33420 fis, clone BRACE2020028		478.16	420.1	470.42	561.48	732.8	688.8	1.45	0.049826
neurocalcin delta /// neurocalcin delta	83988	303.94	234.04	262.68	353.34	418.11	376.52	1.44	0.02062
major histocompatibility complex, class II, D	3113	688.18	528.07	620.6	869.14	879.18	900.34	1.44	0.01786
FYVE and coiled-coil domain containing 1	79443	550.65	527	621	833.42	821.75	798	1.44	0.006162
MRS2-like, magnesium homeostasis factor (	57380	491.95	443.88	435.7	595.86	679.91	693.87	1.44	0.013012
family with sequence similarity 91, member /	57234	436.42	436.86	477.56	671.14	632.47	643.87	1.44	0.000966
carbonic anhydrase XIII	377677	349.26	361.82	384.01	459.88	593.03	527.16	1.44	0.045282
exostoses (multiple) 2	2132	1512.89	1503.48	1670.18	2335.66	2316.57	2074.29	1.43	0.006151
roundabout, axon guidance receptor, homol	6091	2766.82	2632.07	3032.07	3849.09	4188.99	3966.86	1.43	0.002493
chromosome 20 open reading frame 23	55614	344.37	256.47	299.48	452.91	389.59	439.36	1.43	0.019336
membrane-associated ring finger (C3HC4) 5	54708	680.56	572.75	647.34	823.66	954.22	930.87	1.43	0.014547
similar to RIKEN cDNA 1500009M05 gene	493856	1211.11	1013.14	981.95	1589.01	1627.73	1355.25	1.43	0.016521
Hypothetical protein LOC125150	125150	798.9	823.4	733.43	1109.64	1104.88	1132.42	1.43	0.001903
Opsin 1 (cone pigments), short-wave-sensiti	611	1263.78	1444.23	1574.73	1924.98	2114.25	2063.72	1.43	0.013593
ARP2 actin-related protein 2 homolog (yeas	10097	8435.13	7109.31	8300.81	11176.06	10561.25	12050.82	1.42	0.00726
KIAA0241	23080	264.59	231.6	278.82	351.7	380.2	369.37	1.42	0.006046

ABI gene family, member 3 (NESH) binding	25890	2363.45	3261.94	3139.22	4224.78	4079.53	4157.84	1.42	0.046143
methyltransferase like 4	64863	343.64	307.32	341.1	425.07	511.19	481.87	1.42	0.023347
programmed cell death 6	10016	1160.61	1001.71	1013.98	1414.78	1540.41	1523.07	1.42	0.003959
SH3KBP1 binding protein 1	92799	492.44	501.78	567.36	706.96	782.74	716.77	1.41	0.005988
Keratin associated protein 4-7	85287	1577.56	1155.36	1463.05	1929.44	2020.47	1968.91	1.41	0.035273
Protein phosphatase 4, regulatory subunit 2	151987	264.06	311.83	303.9	382.97	420.51	439.16	1.41	0.009445
chondroitin sulfate proteoglycan 2 (versican)	1462	8482.84	8242.12	8043.38	11497.42	11181.35	11946.47	1.4	0.001052
chromosome 12 open reading frame 38	79867	596.99	549.71	498.95	861.7	742.32	722.9	1.4	0.024529
CDK5 regulatory subunit associated protein	54901	287.88	250.59	255.91	381.23	362.26	369.05	1.4	0.004211
CDNA FLJ11754 fis, clone HEMBA1005588		296.83	295.24	299.54	396.77	380.6	469.11	1.4	0.047612
host cell factor C1 regulator 1 (XPO1 depen	54985	1190.82	1307.6	1161.55	1936.95	1559.35	1607.16	1.4	0.039372
G elongation factor, mitochondrial 1	85476	2422.71	2166.96	2381.13	3037.62	3462.67	3243.02	1.4	0.005888
Fraser syndrome 1	80144	869.9	799.57	1010.75	1363.01	1136.03	1272.57	1.4	0.018
poly (ADP-ribose) polymerase family, memb	84875	504.82	505.68	514.12	786.18	690.6	667.72	1.4	0.020711
KIAA1345 protein	57545	377.81	358.49	432.81	496.38	573.67	561.33	1.4	0.015549
chondroitin sulfate proteoglycan 2 (versican)	1462	3236.17	3587.56	3967.33	4783.41	4806.29	5414.9	1.39	0.010559
UPF3 regulator of nonsense transcripts hom	65110	1365.92	1552.61	1465.53	2050.68	2028.66	2005.25	1.39	0.004845
Full-length cDNA clone CS0DI001YP15 of Placenta Cot 25-noi		288.75	325.25	331.43	387.53	460.98	463.5	1.39	0.028494
collagen, type XXVII, alpha 1	85301	711.87	746.72	677.93	960.07	1029.46	962.17	1.39	0.003882
CXYorf1-related protein /// family with seque	375690 /// 653440	937.63	802.41	785.12	1275.84	1123.67	1114.94	1.39	0.012665
WD repeat domain, phosphoinositide interac	26100	444.95	386.16	452.98	610.33	594.97	586.56	1.39	0.006692
UPF3 regulator of nonsense transcripts hom	65110	3031.19	3359.89	3614.47	4616.95	4659.76	4482.15	1.38	0.008422
hypothetical protein FLJ20699	55020	712.69	623.49	614.44	983.72	848.98	857.62	1.38	0.014555
phosphoribosyl transferase domain containi	56952	1802.62	1947.29	1932.87	2385.81	2760.24	2670.08	1.38	0.014843
transmembrane protein with EGF-like and tw	8577	1144	909.04	986.67	1504.22	1328.24	1346.7	1.37	0.017091
laminin, alpha 2 (merosin, congenital muscu	3908	510.92	604.32	623.26	846.57	747.39	793.33	1.37	0.012806
RUN and FYVE domain containing 1	80230	2068.99	1804.75	2157.72	2749.2	2921.68	2571.13	1.37	0.009338
glucosaminyl (N-acetyl) transferase 1, core 2	2650	1071.78	973.1	984.41	1234.86	1400.67	1484.97	1.36	0.029365
fatty acid desaturase 1	3992	2183.67	2069.66	2081.59	2960.14	3016.82	2659.74	1.36	0.01296
gb:AU158251 /DB_XREF=gi:11019772 /DB_XREF=AU158251		320.18	352.8	358.94	483.49	446.82	473.6	1.36	0.007701
misshapen-like kinase 1 (zebrafish)	50488	398.96	338.73	381.35	485.26	545.28	496.49	1.36	0.009859
Ribosomal protein S11	6205	888.17	783.62	793.8	1047.46	1127.96	1157.91	1.36	0.005683
carbonyl reductase 4	84869	525.8	451.29	463.41	652.47	640.27	658.6	1.36	0.007948
acireductone dioxygenase 1	55256	3562.58	4061.21	4130.03	5115.61	5321.61	5525.57	1.36	0.00611
leucine rich repeat containing 8 family, mem	55144	1572.11	1349.49	1418.3	1830.05	2114.02	1938.55	1.36	0.012658
HSPC157 protein	29092	492.62	478.71	467.45	689.98	654.62	622.69	1.36	0.005091
lysyl oxidase-like 3	84695	857.45	913.53	886.41	1074.38	1325.08	1212.16	1.36	0.041083
galactosylceramidase	2581	834.54	798.08	875.31	1189.07	1149.94	1063.15	1.35	0.006105
zinc finger, FYVE domain containing 26	23503	360.75	368.88	416.83	533.05	500.11	515.19	1.35	0.01056
homeobox containing 1	79618	524.07	458.28	445.65	652.98	634.98	647.51	1.35	0.010614
MANSC domain containing 1	54682	373.75	329.54	345.47	473.17	487.26	455.45	1.35	0.014676
chondroitin sulfate proteoglycan 2 (versican)	1462	9285.6	10025.32	10245.4	12140.91	13813.67	13804.81	1.35	0.013686
hypothetical protein MGC16037	84973	2360.86	2333.97	2197.23	3088.25	3098.63	3125.04	1.35	0.001388
homeobox B8	3218	604.14	664.89	662.45	861.87	845.45	887.64	1.35	0.005501
chromosome 9 open reading frame 30	91283	4090.61	3708.59	3523.68	5220.55	5096.19	4947.83	1.35	0.005724
caspase 9, apoptosis-related cysteine peptic	842	428.31	490.39	459.2	604	601.65	638.24	1.34	0.007173
olfactomedin-like 2B	25903	1714.83	1625.65	1654.43	2324.48	2272.73	2089.08	1.34	0.012884

transmembrane protein 50A	23585	4313.49	4148.18	4243.56	5881.08	5600.15	5670.6	1.34	0.000992
chromosome 20 open reading frame 44	55245	655.37	636.92	688.89	947.06	870.11	846.06	1.34	0.008945
STEAP family member 3	55240	3363.1	2710.2	3004.7	3888.84	4141.31	4166.14	1.34	0.01608
ADAM metallopeptidase domain 12 (meltrin	8038	2587.09	2449.5	2528.53	3520.05	3328.38	3178.35	1.33	0.00639
transmembrane protein 66	51669	4626.96	5013.39	5091.06	6209.55	6763.55	6523.29	1.32	0.003859
chromosome 1 open reading frame 57	84284	1059.94	1044.48	1127.42	1490.24	1391.24	1390.53	1.32	0.002173
HIR histone cell cycle regulation defective h	7290	332.57	312.97	307.9	414.44	428.29	414.75	1.32	0.006084
aldehyde oxidase 1	316	1673.47	1530.08	1493.59	2085.75	2094.13	1963.64	1.31	0.003465
hypothetical protein FLJ20186	54849	886.15	950	1015.8	1231.9	1226.76	1273.4	1.31	0.006842
COBW domain containing 6	644019	4139.67	4515.5	4422.78	5534.41	5582.07	5957.48	1.31	0.00308
Hypothetical protein FLJ40432	151195	811.54	837.82	908.52	1073	1149.98	1136.83	1.31	0.004891
transmembrane 9 superfamily protein memb	9777	614.79	614.98	646.53	785.87	812.42	814.08	1.29	0.001472
transcription factor-like 5 (basic helix-loop-h	10732	1511.45	1593.33	1570.97	1263.1	1226.06	1215.41	-1.27	0.002515
gb:AL121916 /DB_XREF=gi:7406639 /FEA=DNA_2 /CNT=1 /T		12049.81	11469.98	11725.38	9238.12	9301.56	9303.43	-1.27	0.002166
RalA binding protein 1	10928	478.43	491.77	511.22	378.84	381.27	389.3	-1.29	0.006104
cell division cycle 2-like 1 (PITSLRE proteins	984 /// 985	1680.62	1545.09	1544.84	1235.19	1278.68	1150.19	-1.3	0.005446
tryptophanyl tRNA synthetase 2 (mitochondr	10352	507.36	551.75	526.75	395.56	413.4	411.35	-1.3	0.004913
gb:AI200538 /DB_XREF=gi:3753144 /DB_XREF=qf93e08.x1 /		475.55	469.09	454.37	359.22	351.24	353.82	-1.31	0.005048
cAMP responsive element binding protein-lik	1388	596.76	653.56	609.61	491.89	442.77	485.36	-1.31	0.006854
tripartite motif-containing 4	89122	1126.42	1157.11	1209.9	875.82	855.45	921.05	-1.32	0.005506
SMAD, mothers against DPP homolog 5 (Dr	4090	3157.68	3165.4	3004.37	2430.1	2168.79	2493.23	-1.32	0.005817
SLIT-ROBO Rho GTPase activating protein	23380	1423.85	1327.45	1328.83	1010.52	970.3	1103.51	-1.32	0.004031
GINS complex subunit 4 (Sld5 homolog) /// (	84296	979.82	905.09	886.24	680.94	670.11	716.93	-1.33	0.008985
UDP-Gal:betaGlcNAc beta 1,4- galactosyltra	9334	2325.42	2133.95	2153.66	1672.39	1704.65	1545.89	-1.34	0.003506
prostaglandin-endoperoxide synthase 1 (pro	5742	665.89	645.69	709.67	498.77	478.51	530.56	-1.34	0.004449
Similar to SLIT-ROBO Rho GTPase-activati	653464	1631.75	1515.2	1575.77	1176.43	1130.44	1228.51	-1.34	0.00108
succinate dehydrogenase complex, subunit	255812	901.66	801.68	786.73	597.3	619.55	627.78	-1.35	0.017611
laminin, alpha 4	3910	4632.23	5021.62	5475.92	3848.52	3472.58	3887.85	-1.36	0.020434
neuroblastoma, suppression of tumorigenici	4681	6663.11	7669.73	7636.9	5719.28	5207.27	5265.67	-1.36	0.0157
neuroblastoma, suppression of tumorigenici	4681	4788.42	5413.93	5515.53	3990.23	3697.81	3780.46	-1.37	0.015888
serine palmitoyltransferase, long chain base	9517	933.01	832.87	871.14	659.01	630.98	640.83	-1.37	0.006313
Transcribed locus		2352.67	2436.6	2258.1	1838.15	1640.01	1664.31	-1.37	0.001997
carbonic anhydrase XII	771	605.92	663.66	715.91	480.46	445.2	517.3	-1.38	0.015149
cell division cycle 42 (GTP binding protein, 2	998	5260.65	5012.44	4825.68	3687.91	3319.29	3896.32	-1.38	0.009496
heme binding protein 2	23593	3433.27	3839.79	3675.09	2853.49	2421.13	2606.48	-1.39	0.007149
histone 1, H2bi	8346	834.85	759.54	799.04	489.66	652.79	578.9	-1.39	0.02988
TIP41, TOR signalling pathway regulator-like	261726	2029.67	1959.46	2261.39	1407.35	1663.71	1418.95	-1.39	0.014709
layilin	143903	7229.64	8063.11	7608.96	5448.12	5464.52	5535.92	-1.39	0.008611
eukaryotic translation initiation factor 2 alpha	440275	401.59	355.71	375.66	239.87	289.21	276.26	-1.39	0.013058
fibroblast growth factor 5	2250	2119.86	1873.7	2060.63	1232.24	1570.6	1515.03	-1.4	0.01531
cell division cycle 2-like 1 (PITSLRE proteins	984 /// 985	450.3	427.87	458.67	317.91	300.33	343.13	-1.4	0.006673
peptidylprolyl isomerase (cyclophilin)-like 3 /	53938	2776.79	2876.06	2807.6	1973.77	2032.27	2037.74	-1.4	0.000225
microphthalmia-associated transcription fact	4286	392.08	342.93	368.45	268.32	247.55	272.64	-1.4	0.007313
SLIT-ROBO Rho GTPase activating protein	57522	1737.67	1662.72	1717.36	1288.51	1099.08	1279.75	-1.4	0.006405
Transcribed locus		1909.94	1831.02	2121.71	1424.68	1364.7	1385.38	-1.41	0.024146
early growth response 1	1958	425.89	350.61	348.7	266.67	245.65	268.26	-1.42	0.042997
frizzled homolog 7 (Drosophila)	8324	1776.4	1863.71	1888.52	1456.17	1095.55	1355.63	-1.42	0.021671

milk fat globule-EGF factor 8 protein	4240	586.35	710.84	689.69	448.65	477.3	474.51	-1.42	0.031232
phosphatidylinositol glycan, class Z	80235	356.12	344.11	368.98	268.23	242.85	247.21	-1.42	0.004608
vascular endothelial growth factor C	7424	10026.03	8982.78	10516.89	6772.93	7402.29	6609.17	-1.43	0.008965
serine racemase	63826	902.71	887.96	935.5	579.16	673.22	661.35	-1.43	0.005135
leucine zipper protein FKSG14	64105	3405.75	2775.01	3003.97	1889.7	2453.55	2066.59	-1.43	0.024073
autocrine motility factor receptor	267	3275.88	2855.25	2848.8	2357.65	1990.61	1894.75	-1.44	0.010845
kelch domain containing 4	54758	432.06	358.77	357.36	270.97	287.9	233.41	-1.44	0.025123
clusterin	1191	540.33	468.32	460.02	304.47	316.75	389.97	-1.45	0.018504
gb:AV695800 /DB_XREF=gi:10297663 /DB_XREF=AV695800		727.27	658.43	690.14	454.87	465.97	498.63	-1.45	0.002765
Transcribed locus		371.01	384.39	427.34	242.98	270.1	299.84	-1.45	0.018729
EP400 N-terminal like	347918	495.46	523.86	567.47	294.31	401.76	394.28	-1.45	0.03214
capping protein (actin filament), gelsolin-like	822	2218.54	2346.14	2356.56	1595.86	1486.45	1603.21	-1.47	0.000783
erythrocyte membrane protein band 4.1 like	57669	535.09	474.62	498.64	359.38	349.14	326.53	-1.47	0.004308
TIP41, TOR signalling pathway regulator-like	261726	2059.23	2242.83	2349.21	1423.23	1667.52	1460.63	-1.47	0.005872
histone 1, H2bf	8343	1348.8	1368.98	1501.67	712.85	1069.42	1064.41	-1.48	0.046056
collagen, type IV, alpha 1	1282	1141.61	1158.96	1299.84	614.07	931.36	873.7	-1.49	0.041319
son of sevenless homolog 1 (Drosophila)	6654	368.08	338.09	323.01	211.19	222.14	250.15	-1.49	0.011969
G protein-coupled receptor 68	8111	1145.04	1182.88	1217.62	887.06	743.88	745.39	-1.49	0.005015
hypothetical protein FLJ20444	403323	362.47	385.5	317.96	253.41	245.88	220.38	-1.49	0.015638
brain-derived neurotrophic factor	627	548.73	472.96	536.6	308.8	378.7	357.6	-1.49	0.007466
zinc finger, MYM-type 6	9204	1090.81	1431.72	1241.55	878.57	738.02	886.77	-1.5	0.037908
histone 1, H2be	8344	1383.26	1057.71	1093.35	645.86	845.01	841.98	-1.51	0.03927
hypothetical protein LOC339803	339803	969.78	1316.29	1109.51	616.72	793.31	832.33	-1.51	0.045696
paired box gene 3 (Waardenburg syndrome	5077	383.59	427.86	429.97	284.64	252.51	283.71	-1.51	0.00507
fibroblast growth factor 5	2250	1601.82	1523.59	1445.02	852.83	1029.43	1121.04	-1.52	0.009598
DALR anticodon binding domain containing	55152	1107.23	1248.14	1207.82	842.17	768.36	744.56	-1.52	0.004694
CGI-96 protein	27341	2592.9	2600.89	2674.15	1575.57	1907.9	1704.7	-1.52	0.007628
polycystic kidney disease 1-like 2	114780	333.3	288.2	263.73	200.07	186.23	194.37	-1.52	0.026389
chromosome 1 open reading frame 166	79594	1087.85	1108.1	1042.2	758.41	680.2	687.7	-1.53	0.000999
Transcribed locus		303.99	294.77	284.58	181.19	195.65	198.34	-1.53	0.002446
methylthioadenosine phosphorylase	4507	320.8	305.13	296.85	185.26	165.95	244.35	-1.54	0.035708
zinc finger, AN1-type domain 2A	90637	2128.4	2373	2219.82	1310.86	1581.16	1487.84	-1.54	0.002932
pyruvate dehydrogenase phosphatase regul	55066	1245.97	1379.61	1488.49	847.63	940.41	873.47	-1.55	0.012365
epithelial membrane protein 2	2013	3785.43	2966.3	2954.81	2173.31	1993.18	2093.75	-1.55	0.047431
importin 8	10526	4264.37	3698.23	4481.74	2383.22	2960.43	2709.52	-1.55	0.008904
D-2-hydroxyglutarate dehydrogenase /// sim 257054 /// 653477		612.07	603.4	595.89	394.43	385.85	392.59	-1.55	0.000367
SEC22 vesicle trafficking protein homolog C	9117	1378.42	1468.8	1434.87	932.09	937.83	855.98	-1.56	0.000502
Poly(A) binding protein, cytoplasmic 1	26986	588.26	542.74	449.85	339.74	314.23	354.18	-1.56	0.034559
Homo sapiens, clone IMAGE:4863312, mRNA		281.54	374.68	337.17	201.03	216.15	218.26	-1.56	0.043787
nuclear factor, interleukin 3 regulated	4783	2082.16	2239.26	2190.24	1584.22	1175.19	1384.31	-1.57	0.010363
stanniocalcin 1	6781	589.23	661.47	699.27	382.88	419.37	438.04	-1.57	0.00833
acyltransferase like 1	54947	267.73	357.4	295.35	184.93	177.69	218.84	-1.57	0.047603
Similar to SLIT-ROBO Rho GTPase-activati	653464	1394.47	1873.98	1788.41	933.73	1117.83	1157.07	-1.58	0.040885
Hypothetical protein similar to KIAA0187 ge	96610	757.62	756.3	867.58	426.43	524.53	548.25	-1.58	0.007641
gb:AI220472 /DB_XREF=gi:3802675 /DB_XREF=qg43e04.x1		849.71	702.03	728.3	409.71	522.53	505.05	-1.58	0.010476
CGI-96 protein	27341	787.71	739.93	719.93	447.75	465.38	482.21	-1.6	0.001017
secreted phosphoprotein 1 (osteopontin, bo	6696	1123.67	886.51	971.74	447.62	702.1	703.95	-1.6	0.031505

cell division cycle associated 7 /// cell divisio	83879	2217.57	1678.59	1908.34	856.98	1492.79	1268.56	-1.6	0.043287
pyruvate dehydrogenase phosphatase regul	55066	1101.6	1168.83	1061.42	761.94	630.63	692.48	-1.6	0.001767
hypothetical protein LOC645644	645644	369.55	462.15	411.56	229.71	242.41	305.74	-1.6	0.015941
Dedicator of cytokinesis 2	1794	3009.65	3112.92	3775.93	1401.02	2428.51	2359.8	-1.6	0.046461
acid phosphatase 1, soluble	52	2314.24	2225.94	2081.19	1408	1288.79	1417.58	-1.61	0.001189
aryl hydrocarbon receptor	196	3685.39	3674.68	3722.48	2777.01	2031.88	2085.55	-1.61	0.024601
stanniocalcin 1	6781	899.06	751.31	943.96	493.71	593.87	526.68	-1.61	0.015616
microfibrillar associated protein 5	8076	804.64	1052.08	1119	450.8	720.79	674.67	-1.61	0.046866
methylthioadenosine phosphorylase	4507	606.71	512.39	516.9	309.92	316.69	387.4	-1.61	0.009414
guanylate binding protein 3	2635	2169.24	2503.5	2492.68	1459.7	1468.57	1533.06	-1.61	0.011461
microfibrillar associated protein 5	8076	2881.43	3029.48	2978.5	1562.5	1984.95	1951.21	-1.62	0.008784
chromosome 20 open reading frame 100	84969	1558.42	1193.29	1329.19	818.7	860.84	815.29	-1.63	0.034019
hypothetical protein LOC647322	647322	395.03	489.63	438.47	247.47	286.43	271.23	-1.63	0.018959
clusterin associated protein 1	23059	432.58	469.93	498.31	256.65	305.14	294.84	-1.64	0.004276
epithelial membrane protein 2	2013	1782.69	1422.05	1485.45	1031.23	942.82	910.47	-1.64	0.023855
CDNA FLJ30156 fis, clone BRACE2000487		605.76	548.36	511.93	351.79	329.46	335.71	-1.64	0.00993
anthrax toxin receptor 1	84168	531.95	544.88	540.58	401.25	304.18	277.12	-1.65	0.023701
serine incorporator 2	347735	1847.88	1505.18	1574.67	1035.34	969.54	969.8	-1.66	0.018161
chromosome 6 open reading frame 139	55166	1258.18	1119.91	1184.15	490.72	821.49	814.02	-1.67	0.037056
Peroxisomal LON protease like	83752	278.33	381.58	374.66	197.14	199.51	228.31	-1.67	0.042224
phenylalanine-tRNA synthetase-like, beta su	10056	395.2	401.5	401.85	217.24	248.19	249.65	-1.68	0.0009
hypothetical protein MGC3207	84245	1501.81	1886.6	1835.07	962.88	1038.42	1090.92	-1.69	0.020811
suprabasin	374897	457.12	521.15	469.29	368.51	231.56	259.1	-1.69	0.024455
microfibrillar associated protein 5	8076	1150.71	1355.29	1479.37	619.81	864.45	859.23	-1.7	0.014486
tetraspanin 18	90139	210.17	286.44	264.94	170.47	149.03	135.48	-1.7	0.033493
major histocompatibility complex, class I, F	3134	1151.2	1237.52	1070.75	795.6	577.33	644.77	-1.71	0.008873
gb:BE271180 /DB_XREF=gi:9144831 /DB_XREF=600943914I		441.67	382.96	444.56	232.35	264.7	236.79	-1.71	0.007044
coagulation factor X	2159	273.2	345.05	326.91	240.63	155.52	151.76	-1.72	0.026438
pleckstrin homology domain containing, fam	26030	510.55	393.98	428.95	237.19	271.36	252.08	-1.73	0.017772
lumican	4060	6695.33	7640.2	6828.04	4389.49	3611.99	4200.8	-1.74	0.003379
coagulation factor II (thrombin) receptor	2149	1245.5	1298.14	1475.2	629.64	794.48	867.5	-1.74	0.006165
CDNA clone IMAGE:5261717		259.62	254.31	211.9	148.3	118.05	152.16	-1.74	0.008351
ras-related C3 botulinum toxin substrate 2 (r	5880	1400.15	1851.92	1917.44	674.37	1163.87	1109.14	-1.75	0.033652
hypothetical LOC388610	388610	10223.63	13885.63	13466.16	4557.12	7814.81	9096.74	-1.75	0.043567
hypothetical protein LOC150837	150837	542.75	689.36	715.36	394.89	341.16	379.09	-1.75	0.025686
gb:AI763262 /DB_XREF=gi:5178929 /DB_XREF=wi66c04.x1 /		554.84	502.43	544.84	287.24	312.74	315.03	-1.75	0.000831
KIAA0265 protein	23008	1019.7	1097.7	1063.75	672.87	572.25	550.71	-1.76	0.007915
Serine hydrolase-like 2	253190	428.47	371.62	362.86	173.95	255.42	222.14	-1.77	0.008878
collagen, type XVIII, alpha 1	80781	1466.19	1893.84	1815.74	861.46	974.32	1066.16	-1.78	0.017281
major histocompatibility complex, class II, D	3115	1077.6	1074.49	1152.12	569.65	632.59	643.89	-1.79	0.019474
hypothetical protein MGC9913	386759	990	1053.27	959.17	604.39	554.31	528.22	-1.79	0.000804
gb:AI797678 /DB_XREF=gi:5363150 /DB_XREF=we90c08.x1		757.37	613.05	575.24	284.53	396.03	395.85	-1.8	0.016638
trafficking protein particle complex 4	51399	6080.79	6361.38	6052.05	3051.72	3708.18	3428.41	-1.81	0.000705
nuclear RNA export factor 3	56000	289.89	321.79	400.19	146.96	214.67	197.05	-1.81	0.025215
leucine rich repeat containing 2	79442	430.41	545.19	536.94	242.65	286.42	306.65	-1.81	0.013695
T-box 1	6899	1799.91	1452.6	1644.22	1024.71	811.72	842.74	-1.83	0.00541
uveal autoantigen with coiled-coil domains a	55075	248.02	267.85	309.88	128.18	170.87	151.07	-1.83	0.009938

Homo sapiens, clone IMAGE:5019307, mRNA		456.26	545.16	520.57	251.69	283.42	293.82	-1.83	0.005761
insulin-like growth factor 1 receptor	3480	976.52	1218.91	957.26	736.31	374.92	606.26	-1.84	0.025977
Chromosome X open reading frame 33	139322	251.08	310.28	348.42	141.02	176.08	174.41	-1.85	0.028871
Hypothetical gene supported by BC041875;	400642	369.91	331.35	286.04	193.32	164.47	171.64	-1.86	0.013096
kelch-like 18 (Drosophila)	23276	238.39	281.11	263.61	123.13	148.08	149.44	-1.86	0.003901
nerve growth factor, beta polypeptide	4803	338.61	430.15	401.62	189.17	231.68	202.79	-1.87	0.011191
hypothetical gene supported by AK128660	400566	541.31	490.56	503.39	242.82	285.75	282.58	-1.87	0.0012
glucuronidase, beta-like 2	375513	262.01	252.39	260.89	141.52	139.29	135.25	-1.87	0.003328
trafficking protein particle complex 4	51399	5170.66	5763.1	5677.11	2836.78	2969.6	3050.96	-1.88	0.00274
major histocompatibility complex, class I, C	3107	7451.37	6708.48	6588.17	4705.09	3188	3189.63	-1.89	0.011622
sperm equatorial segment protein 1	246777	591.31	602.45	607.24	340.57	310.99	307.7	-1.89	0.000113
serine palmitoyltransferase, long chain base	10558	448.7	567.43	536.4	216.9	302.36	300.33	-1.89	0.008824
cyclin-dependent kinase 6	1021	1962.45	2500.25	2121.13	1085.57	1011.37	1402.04	-1.9	0.009941
phosphatase and actin regulator 3	116154	443.32	352.97	376.71	289.51	145.52	171.27	-1.93	0.030429
tumor necrosis factor receptor superfamily, r	8797	348.3	296.42	255.69	177.78	138.14	150.85	-1.93	0.018646
HRAS-like suppressor 3	11145	485.14	610.8	637.19	252.95	322.66	309.6	-1.94	0.014167
leucine rich repeat containing 15	131578	12309.32	12922.81	14282.51	5312.95	7788.05	7086.86	-1.96	0.003584
gb:AA021163 /DB_XREF=gi:1484898 /DB_XREF=ze65e10.s1		415.42	378.58	352.56	198.27	182.47	193.69	-1.96	0.003682
chromosome 21 open reading frame 7	56911	1573.98	1242.29	1215.28	614.82	759.13	661.2	-1.98	0.017805
neurobeachin	26960	526.85	631.39	632.44	253.27	315.08	336.23	-1.99	0.005346
amyloid beta (A4) precursor protein-binding,	54518	1504.01	1248.33	1464.22	624.05	747.85	733.5	-2	0.003838
Transcribed locus		218.09	324.05	307.8	123.83	148.86	150.31	-2.01	0.040761
thioredoxin interacting protein	10628	983.04	878.61	847.9	527.92	418.8	389.62	-2.03	0.002182
neurobeachin	26960	256.36	317.19	317.91	120.65	139.91	179.63	-2.03	0.008345
gb:BF063164 /DB_XREF=gi:10822074 /DB_XREF=7h84e05.x		255.78	319.43	372.39	83.46	186.4	188.79	-2.09	0.034815
Splicing factor, arginine/serine-rich 3	6428	279	367.49	322.46	147.45	172.23	142.13	-2.1	0.015386
creatine kinase, brain	1152	1212.08	963	1010.3	647.4	404.32	451.53	-2.12	0.006349
early growth response 1	1958	1493.35	1520.77	1458.22	714.3	629.91	749.05	-2.14	0.000165
KIAA1913	114801	4032.21	3255.01	4139.88	1730.11	1947.48	1646.52	-2.14	0.011555
ets variant gene 1	2115	980.56	765.6	797.27	395.51	387.79	396.67	-2.15	0.018261
UDP-Gal:betaGlcNAc beta 1,4- galactosyltra	9334	2748.4	3061.32	2862.93	1469.69	1126.38	1405.51	-2.17	0.000512
hypothetical protein LOC255480	255480	1284.98	1000.8	1110.29	534.74	482.09	481.76	-2.25	0.011836
thioredoxin interacting protein	10628	382.75	350.08	277.35	181.06	111.92	150.62	-2.26	0.012943
heart and neural crest derivatives expressec	9464	359.79	296.08	328.66	171.37	134.83	120.99	-2.28	0.002429
Transcribed locus		369.21	376.15	372.31	139.01	153.44	184.11	-2.33	0.003697
hypothetical protein MGC9913	386759	1219.23	1404.42	1360.81	738.63	469.09	526.65	-2.33	0.003279
complement factor D (adipsin)	1675		445.21	393.43	277.95	137.69	124.14	-2.35	0.021805
collagen, type XV, alpha 1	1306	419.74	632.55	632.61	278.63	218.08	212.82	-2.37	0.037245
opioid growth factor receptor	11054	486.03	475.73	488.62	232.38	177.17	196.5	-2.38	0.001628
beta 1,3-galactosyltransferase-like	145173	1155.78	1122.01	1085.66	521.35	457.41	438.55	-2.38	0.000152
SEC31-like 1 (S. cerevisiae)	22872	1382.56	1345.35	1343.18	562.69	543.45	569.58	-2.43	0.000021
nicotinate phosphoribosyltransferase domaii	93100	483.65	664.73	634.31	233.99	259.91	236.24	-2.44	0.01541
leucine rich repeat containing 2	79442	495.66	519.11	605.29	182.31	252.52	217.56	-2.48	0.002459
prostaglandin E receptor 2 (subtype EP2), 5	5732	326.89	491.07	520.42	139.37	189.51	205.94	-2.5	0.037269
hypothetical protein LOC255480	255480	1092.41	874.18	915.53	422.39	350.79	352.09	-2.57	0.006617
Forkhead box C2 (MFH-1, mesenchyme forl	2303	288.14	346.56	408.48	89.72	153.87	134.12	-2.71	0.011195
T-box 5	6910	1562.87	1449.41	1516.98	495.55	574.48	577.43	-2.74	0.000051

mesoderm specific transcript homolog (mou	4232	297.37	416.5	443.21	99.17	162.11	154.91	-2.76	0.018132
calponin 1, basic, smooth muscle	1264	1319.26	1634.92	1694.79	448.53	672.72	547.73	-2.77	0.004264
zona pellucida glycoprotein 3 (sperm recept	22932 /// 7784	741.99	732.21	721.64	246.02	273.51	247.84	-2.86	0.000122
arylsulfatase A	410	370.4	351.75	383.83	143.37	127.65	117.01	-2.87	0.000225
Similar to TFIIH basal transcription factor co	653249	145.54	142.08	181.96	39.43	61.39	56.81	-2.95	0.005024
chromosome 8 open reading frame 59	401466	2427.8	2279.78	2187.22	755.36	796.81	762.47	-2.97	0.000919
hypothetical protein MGC24665	116028	1684.63	1417.37	1488.88	419.49	562.15	533.28	-3.01	0.001232
major histocompatibility complex, class II, D	3112	324.34	395.51	369.02	105.64	133.86	112.86	-3.04	0.001405
T-box 5	6910	457.94	403.68	328.68	136.88	101.35	152.77	-3.04	0.008743
heat shock 70kDa protein 2	3306	6200.85	5558.91	5755.69	2558.2	1496.54	1673.56	-3.06	0.001294
transmembrane 4 L six family member 1	4071	963.71	911.34	892.44	244.5	333.33	325.48	-3.07	0.000147
transmembrane 4 L six family member 1	4071	542.85	511.07	482.14	135.84	186.41	171.52	-3.09	0.000542
zona pellucida glycoprotein 3 (sperm recept	22932 /// 7784	594.98	619.24	576.9	167.44	208.01	202.61	-3.09	0.000091
cytochrome P450, family 1, subfamily B, pol	1545	4093.43	5983.25	5005.91	2499.62	761.03	1564.94	-3.13	0.010402
NK3 transcription factor related, locus 1 (Dro	4824	2366.21	1957.38	2332.42	584.81	737.92	740.59	-3.23	0.002723
KIAA0265 protein	23008	1053.98	1097.45	1116.46	385.18	286.88	332.61	-3.25	0.000052
insulin-like growth factor binding protein 5	3488	1485.78	1256.44	1335.43	227.49	507.02	507.78	-3.28	0.002011
CDNA FLJ36559 fis, clone TRACH2009291		186.29	205.99	257.97	30.57	76.75	92.46	-3.28	0.007446
hypothetical LOC388796	388796	3513.11	3377.2	3270.31	893.16	1121.27	1058.36	-3.3	0.000028
cytochrome P450, family 1, subfamily B, pol	1545	2133.54	4067	3657.46	1389.32	616.17	974.94	-3.33	0.046995
cytochrome P450, family 1, subfamily B, pol	1545	4376.66	6604.46	5594.4	2504.56	878.19	1580.09	-3.35	0.011019
transmembrane 4 L six family member 1	4071	371.88	327.69	312.81	75.87	111.95	106.02	-3.42	0.000821
G0/G1switch 2	50486	880.27	1019.64	1104.75	212.94	324.55	329.98	-3.43	0.002007
integrin, beta 8	3696	127.15	203.14	208.16	50.31	48.77	54.99	-3.52	0.037238
Growth hormone regulated TBC protein 1	79774	372.63	287.14	298.48	117.78	78.48	75.41	-3.52	0.004106
MAM domain containing 2	256691	168.73	266.32	287.33	64.17	75.75	54.63	-3.74	0.037063
hypothetical LOC388796	388796	1740.78	1929.98	1832.7	420.21	479.34	479.76	-3.96	0.000261
Protein kinase, cAMP-dependent, regulatory	5575	303.4	358.05	339.81	93.25	79.04	80.84	-3.99	0.000708
solute carrier family 35, member E2	9906	101.82	169.08	137.86	30.89	33.44	35.14	-4.12	0.02924
fibrillin 2 (congenital contractural arachnoda	2201	1580.95	1442.52	1481.89	453.35	285.91	302.76	-4.3	0.000096
cartilage oligomeric matrix protein	1311	1285.1	1816.53	1866.56	509.64	324.96	321.94	-4.34	0.01524
secreted frizzled-related protein 2	6423	1288.19	1198.72	1190.92	277.91	274.62	294.9	-4.34	0.000197
phosphodiesterase 4D interacting protein (m	9659	1769.59	1425.28	1494.07	430.85	349.22	315.21	-4.37	0.001175
secreted frizzled-related protein 2	6423	2360.85	4104.22	3977.92	583.18	481.47	589.53	-6.32	0.034852
latexin	56925	707.22	934.67	926.52	123.87	125.13	146.1	-6.51	0.008643
mab-21-like 1 (C. elegans)	4081	1667.54	1795.89	1851.71	176.01	189.44	158	-10.14	0.000945
